# Insights into the Effect of Catalytic Intratumoral Lactate Depletion on Metabolic Reprogramming and Immune Activation for Antitumoral Activity

**DOI:** 10.1002/advs.202204808

**Published:** 2022-12-07

**Authors:** Junlong Zhao, Zhimin Tian, Shoujie Zhao, Dayun Feng, Zhixiong Guo, Liangzhi Wen, Yejing Zhu, Fenghua Xu, Jun Zhu, Shouzheng Ma, Jie Hu, Tao Jiang, Yongquan Qu, Dongfeng Chen, Lei Liu

**Affiliations:** ^1^ Department of Gastroenterology Daping Hospital Army Medical University Chongqing 400032 P. R. China; ^2^ State Key Laboratory of Cancer Biology Department of Medical Genetics and Development Biology Fourth Military Medical University Xi'an 710032 P. R. China; ^3^ Key Laboratory of Special Functional and Smart Polymer Materials of Ministry of Industry and Information Technology School of Chemistry and Chemical Engineering Northwestern Polytechnical University Xi'an 710072 P. R. China; ^4^ Xi'an People's Hospital (Xi'an Fouth Hospital) Shaanxi Eye Hospital Affiliated Guangren Hospital School of Medicine Xi'an Jiaotong University Xi'an 710004 P. R. China; ^5^ Department of General Surgery Tangdu Hospital Fourth Military Medical University Xi'an 710038 P. R. China; ^6^ Department of Surgery Tangdu Hospital Fourth Military Medical University Xi'an 710038 P. R. China; ^7^ Present address: Department of Gastroenterology Chongqing Key Laboratory of Digestive Malignancies Daping Hospital, Army Medical University (Third Military Medical University) Chongqing 400042 P. R. China

**Keywords:** catalysis, immunotherapy, lactate, metabolism, tumor

## Abstract

Lactate, a characteristic metabolite of the tumor microenvironment (TME), drives immunosuppression and promotes tumor progression. Material‐engineered strategies for intratumoral lactate modulations demonstrate their promise for tumor immunotherapy. However, understanding of the inherent interconnections of material‐enabled lactate regulation, metabolism, and immunity in the TME is scarce. To address this issue, urchin‐like catalysts of the encapsulated Gd‐doped CeO_2_, syrosingopine, and lactate oxidase are used in ZIF‐8 (USL, where U, S, and L represent the urchin‐like Gd‐doped CeO_2_@ZIF‐8, syrosingopine, and lactate oxidase, respectively) and orthotopic tumor models. The instructive relationships of intratumoral lactate depletion, metabolic reprogramming, and immune activation for catalytic immunotherapy of tumors is illustrated. The catalysts efficiently oxidize intratumoral lactate and significantly promote tumor cell apoptosis by in situ‐generated ·OH, thereby reducing glucose supply and inducing mitochondrial damage via lactate depletion, thus reprogramming glycometabolism. Subsequently, such catalytic metabolic reprogramming evokes both local and systemic antitumor immunity by activating M1‐polarizaed macrophages and CD8^+^ T cells, leading to potent antitumor immunity. This study provides valuable mechanistic insights into material‐interfered tumor therapy through intratumoral lactate depletion and consequential connection with metabolic reprogramming and immunity remodeling, which is thought to enhance the efficacy of immunotherapy.

## Graphical abstract

Lactate is the primary tumor immunosuppressive factor limiting the effectiveness of tumor treatment. The authors develop a secure biocatalytic immunoenhancement strategy to boost antitumor immunity by consuming intratumoral lactate and inducing metabolic reprogramming using a nanoreactor. Through the design of this delicate catalytic immunoenhancement strategy, nanoreactors with lactate as a target may open a promising and secure avenue for tumor therapy.

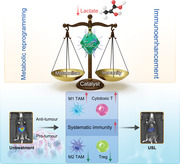

## 1 Introduction

Abnormal enzyme catalytic processes and their products induce metabolic reprogramming in tumors, thereby causing immunosuppression and further failure of tumor therapy.^[^ ^1^, ^2^, ^3^, ^4^ ^]^ The catalytic modulation of metabolites can provide a feasible approach to reprogram metabolism and enhance antitumor immunity in the tumor microenvironment (TME). Many antitumor drugs and inhibitors have been used clinically to influence metabolic enzymes and sequentially tailor metabolites for tumor therapy.^[^ ^5^, ^6^ ^]^ With this in mind, developing an effective biomimetic catalytic strategy to modulate metabolism may be beneficial for tumor therapy, which will also help elucidate the functions and crosstalk mechanisms of tumor metabolism and immunity.

Notably, among the complex metabolic pathways of tumors, the overactivation of glycolysis is a universal hallmark of cancers.^[^ ^7^ ^]^ Lactate, a major metabolite of glycolysis and a biomarker of tumor development, is generally overproduced and accumulated in all types of tumors, inducing an acidic TME and leading to subsequent immunosuppression.^[^ ^8^, ^9^ ^]^ Apart from altering the metabolism to facilitate the proliferation and metastasis of tumor cells, tumor‐derived lactate is the primary factor in immune regulation. Lactate accumulation in the TME accelerates the M2 polarization switch of macrophages, promotes myeloid‐derived suppressor cell (MDSC) generation, represses T cell activation, and inhibits NK cell‐mediated cytotoxicity.^[^ ^7^, ^10^, ^11^, ^12^ ^]^ Accumulated evidence has verified that inhibiting lactate transport modulates immune cell development and represses tumor progression.^[^ ^9^ ^]^ Unfortunately, tumors are systemic diseases that influence the immune system. Antitumor immunity is regulated locally (intratumoral immunity) and globally (systemic immunity). Hence, simultaneously restoring the original antitumor activity of both intratumoral and systemic immunity is of great significance for tumor therapy.^[^ ^13^, ^14^, ^15^ ^]^ However, simply repressing lactate transport with inhibitors cannot eliminate lactate; instead, it causes continuous accumulation of lactate in the TME or in circulation, which results in global metabolic dysregulation and systemic immunosuppression. Therefore, an effective approach that enables lactate elimination is critical for metabolism‐based cancer immunotherapies.^[^ ^16^, ^17^ ^]^

**Figure 1:**
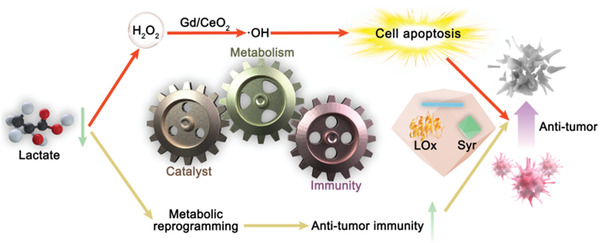
Illustration of the specifically designed catalysts for the catalytic immunotherapy of tumors.

Lactate can be efficiently eliminated in vivo by natural enzymes, such as lactate oxidase (Lox).^[^ ^18^, ^19^, ^20^, ^21^, ^22^, ^23^, ^24^, ^25^ ^]^ Inspired by the universal existence of abundant lactate in tumors, the above recognitions have stimulated our interest in the intratumoral catalytic consumption of lactate and its consequential biological effects against tumors.^[^ ^26^, ^27^, ^28^, ^29^, ^30^, ^31^, ^32^, ^33^, ^34^ ^]^ Herein, we successfully developed a catalytic metabolic reprogramming strategy via intratumoral catalytic elimination of lactate to simultaneously enable both local and systemic immune remodeling and catalytic generation of ·OH for tumor therapy (**Figure** **1**). More importantly, this study focused on how the catalytic metabolic regulation of lactate can help enhance immune function and its impact on related energy metabolism. We dissected the role of catalytic metabolic strategy in the modulation of antitumor immunity into three parts: 1) modulation of immune cells for enhancing intratumoral immunity; 2) influencing immune cell development for the activation of systemic immunity; and 3) inhibition of glycometabolism within the TME to reverse immunosuppression. We examined the regulatory mechanisms that drive metabolic reprogramming by glucose limitation and mitochondrial damage, and discuss how these metabolic pathways further regulate antitumor immunity. The lactate catalytic metabolic reprogramming strategy that regulates immune responses in the TME will provide insights into the complex relationships between metabolism and antitumor immunity. This catalytic metabolic reprogramming strategy with lactate as a target might open a promising and secure avenue for tumor therapy, which could be extended to other characteristic metabolites in the TME.

## 2 Results

### 2.1 Design and Synthesis of Catalysts

**Figure 2:**
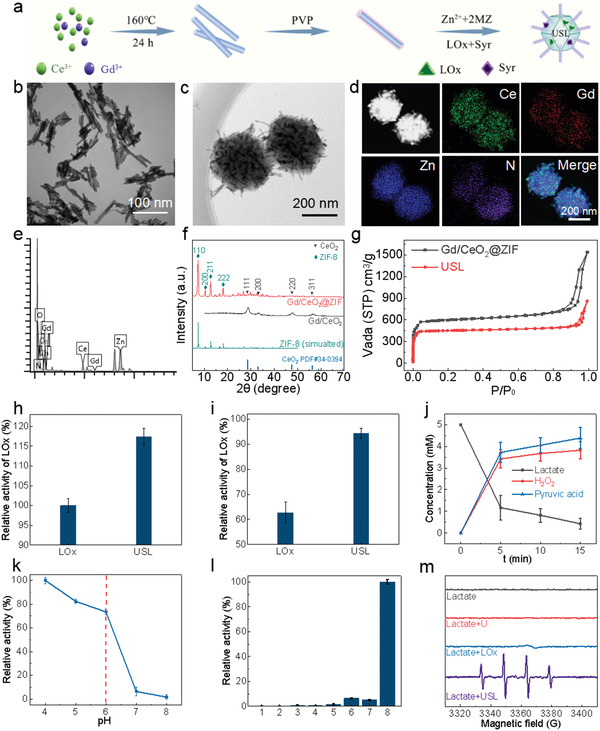
Synthesis, characterization, and catalytic performance of the USL catalysts. a) Synthetic protocol of USL. b) TEM image of Gd/CeO_2_. c,d) TEM images and the elemental mapping of USL. e) The EDS spectrum of USL. f) XRD patterns of Gd/CeO_2_ and Gd/CeO_2_@ZIF. g) N_2_ adsorption profiles of Gd/CeO_2_@ZIF and USL. h) Relative activities of free LOx and USL for lactate oxidation. i) Relative activities of free LOx and USL stored at 37 °C for 14 d. The catalytic activity of the fresh catalysts was set as 100%. The concentrations of free LOx and LOx in USL were controlled at 200 µg of LOx mL^−1^. The reaction conditions were 37 °C for 10 min in PBS buffer (pH 6.0). j) Catalytic profiles of USL by monitoring lactate and products of pyruvic acid and H_2_O_2_. k) Evaluations of the pH‐dependent peroxidase‐like activity of USL with lactate as the substrate. Reaction conditions: Lactate: 5 × 10^−3^ m, USL: 0.1 mg mL^−1^, and TMB: 1.5 × 10^−3^ m. The maximum activity of the catalysts in all measurements was set as 100%. l) Oxidation products of TMB (652 nm) under various conditions in the NaAc buffer. Absorbance at 652 nm of the TMB (1.5 × 10^−3^ m) reaction solutions catalyzed by different catalysts at 37 °C for 10 min (acetate buffer, 20 × 10^−3^ m, pH 6.0). 1: TMB; 2: Lactate + TMB; 3: LOx + TMB; 4: Lactate + LOx + TMB; 5: US + TMB; 6: US + Lactate + TMB; 7: USL +TMB; and 8: USL + Lactate +TMB. m). ESR spectra of the spin trap 5‐tert‐butoxycarbonyl‐5‐methyl‐1‐pyrroline‐N‐oxide‐·OH adduct during incubations under various conditions.

A catalyst of encapsulated LOx, Gd‐doped CeO_2_ nanorods (Gd/CeO_2_), and the monocarboxylate transporter‐4 inhibitor syrosingopine (Syr) within the acidic‐soluble zinc zeolitic imidazolate framework (ZIF‐8) was designed (**Figure** **2**a). ZIF, a common type of metal‐organic framework, serves as a highly advantageous universal carrier of syrosingopine and LOx with a high loading efficacy to enrich the effective components in tumor tissues. In addition, the Gd/CeO_2_ nanorods grow radially from the inner ZIF structure, giving a rough mimicking structure similar to the spikes of a sea urchin. Encouragingly, the spiky protrusions of nanostructures have been reported to enhance cellular uptake due to their biomimetic morphology, which can lead to increased intratumoral USL accumulation to enhance antitumor activity.^[^ ^35^, ^36^ ^]^ Specifically, syrosingopine reduces lactate export from tumors by inhibiting monocarboxylate transporter‐4 (MCT4) activity.^[^ ^37^ ^]^ LOx can effectively oxidize lactate and produce a considerable amount of H_2_O_2_. Meanwhile, endogenous and exogenous H_2_O_2_ are catalyzed to produce hydroxyl radicals under Gd‐doped CeO_2_ (Gd/CeO_2_) peroxidase (POD)‐like activity. Owing to the existence of mixed‐valence states of Ce^3+^ and Ce^4+^ and the existence of oxygen vacancies, CeO_2_ displays excellent enzyme‐like catalytic performance, especially peroxidase‐like activity in weakly acidic environments, indicating their potential as nanozymes for catalytic therapy because of their superior chemical stability and biocompatibility.^[^ ^38^, ^39^, ^40^, ^41^ ^]^ Therefore, we developed a Gd/CeO_2_ nanozyme that delivers outstanding POD‐like performance in weakly acidic environments but inhibits POD‐like activity under normal physiological conditions. Such catalytic behavior suggests the potential use of nanoceria for selective tumor therapy within the TME.^[^ ^42^ ^]^

Initially, crystalline fluorite CeO_2_ and Gd/CeO_2_ nanorods, with lengths of 50–150 nm and diameters of 10–15 nm, were hydrothermally synthesized (Figure 2b and Figure S1, Supporting Information).^[^ ^43^ ^]^ Substitution of Ce^4+^ ions by foreign cations with different ionic radii and oxidation states can change the inherent oxygen vacancy concentration, as well as the surface Ce^3+^ fraction, leading to modulated redox properties of CeO_2_ between Ce^3+^ and Ce^4+^.^[^ ^44^, ^45^ ^]^ The atomic molar ratio of Gd/(Gd+Ce) in Gd/CeO_2_ was 4.3%, as determined by inductively coupled plasma‐mass spectrometry. Based on X‐ray photoelectron spectroscopic profiles (Figure S2, Supporting Information), the Gd/CeO_2_ presented a higher Ce^3+^ fraction of 35.5% than CeO_2_ nanorods (28.3%), which could be attributed to the successful chemical doping of Gd^3+^ in the CeO_2_ lattice. Consequently, the peroxidase‐like activity of Gd/CeO_2_ was 1.8‐times higher than that of CeO_2_ (Figure S3, Supporting Information).^[^ ^44^, ^45^ ^]^ The peroxidase‐like activity of Gd/CeO_2_ is pH‐dependent, similar to that of CeO_2_, and thereby contributes to the subsequent pH‐regulated tumor catalytic therapy.^[^ ^39^, ^40^ ^]^

Next, an urchin‐like USL, in which several Gd/CeO_2_ nanorods, LOx, and Syr were encapsulated by ZIF‐8, was prepared using a wet‐chemical approach.^[^ ^20^, ^46^ ^]^ To illustrate the morphological features and multiple components of the as‐synthesized USL catalysts, various structural characterizations, including transmission electron microscopy (TEM), dark‐field TEM, energy‐dispersive spectrometry (EDS), and elemental mapping, were performed. TEM and dark‐field TEM images clearly showed the urchin‐like structural features of the as‐synthesized USL (Figure 2c,d and Figure S4, Supporting Information), in which Gd/CeO_2_ nanorods were centrally located in the USL to form spiky Gd/CeO_2_ protrusions on the surface of the ZIF‐8 core with an overall nanoreactor size of ≈300 nm. Furthermore, the homogeneous distributions of the Ce, Gd, Zn, and N signals in the USL were confirmed by elemental mapping (Figure 2d). The coexistence of Ce, Gd, Zn, and N signals was further demonstrated by the EDS spectra of the USL (Figure 2e), indicating the successful and dispersive loading of Gd/CeO_2_, Syr, and LOx within the ZIF‐8 framework. The X‐ray diffraction pattern further confirmed the coexistence of the Gd/CeO_2_ nanorods and ZIF in the USL. As shown in Figure 2f, the diffraction peaks at angles of 28.9°, 33.2°, 47.9°, and 56.5° in the (111), (200), (220), and (311) planes, respectively, of the Gd/CeO_2_ nanorods in the XRD patterns can be assigned to fluorite‐structured ceria. In comparison with the XRD pattern of Gd/CeO_2_ and the simulated XRD pattern of ZIF‐8, the XRD spectrum of the Gd/CeO_2_@ZIF samples clearly confirmed the coexistence of the Gd/CeO_2_ and ZIF structures in the composite. The Fourier transform infrared spectroscopy (FT‐IR) spectrum of USL alone displayed characteristic methyl peaks at 1735–1750 and 2865–2875 cm^−1^, further confirming the formation of ZIF‐8 in the catalysts (Figure S5, Supporting Information). Additionally, the characterized vibrations of LOx molecules, including C‐N at 1154 cm^−1^ and 1073 cm^−1^, were observed for the USL, again demonstrating the successful immobilization of LOx in the catalysts (Figure S5, Supporting Information). The Brunauer–Emmett–Teller (BET) area of the USL dropped from 1817 to 1357 m^2^ g^−1^ for Gd/CeO_2_@ZIF due to the incorporation of LOx and Syr in the parent framework (Figure 2g). All characterizations demonstrated the formation of urchin‐like catalysts with the features of encapsulated multiple components of LOx, Gd/CeO_2_, and Syr within ZIF‐8.

Importantly, the radial growth of the Gd/CeO_2_ nanorods in the USL induced the formation of the spiky protrusion configuration of the USL, which has been reported to exhibit superior cellular uptake capability owing to this biomimetic morphology and, thereafter, potentially increase the intratumoral accumulation of USL.^[^ ^47^ ^]^ Compared to Gd/CeO_2_ nanorods, the zeta potential of USL was increased to 37.2 mV due to the presence of the imidazole group (Figure S6a, Supporting Information), which again supports the successful formation of the ZIF‐8 nanostructure. The similar hydrodynamic diameters (275 nm) of USL in water and cell culture medium demonstrated its high stability in the medium (Figure S6b, Supporting Information), which suggests its potential for in vivo and in vitro applications. To investigate the catalytic performance of the USL, various control catalysts, including Gd/CeO_2_@ZIF (nanourchin, U), Syr@nanourchin (US), and LOx@nanourchin (UL), were also synthesized using a similar method, except in the absence of Syr or LOx (Figure S7, Supporting Information).

### 2.2 Catalytic Performance of USL for Lactate Oxidation

As indicated by the Bradford assay and high‐performance liquid chromatography, the amounts of LOx and Syr encapsulated in USL were 135 µg mg^−1^ (13.5% loading) and 113 µg mg^−1^ (11.3% loading), respectively. At the same level as LOx, the USL showed 17.4% higher lactate oxidation activity than free LOx (Figure 2h).^[^ ^46^ ^]^ Severe activity decay was observed for free LOx, with only 62.7% of the activity preserved at 37 °C after 14 d (Figure 2i). Comparatively, 94.2% of the LOx function of the USL was preserved for two weeks under the same conditions. Analysis of catalytic lactate oxidation by LOx and USL showed near‐stoichiometric production of pyruvic acid and H_2_O_2_ at a pH of 6.5 (Figure 2j and Figure S8, Supporting Information). USL showed pH‐dependent activity with a significant ROS release from lactate at pH 6.5 and minimal ROS production at pH ≥7.0 (Figure 2k,l and Figure S9, Supporting Information), indicating its potential for selective catalytic chemotherapy based on lactate generated by tumor metabolism in the weakly acidic TME. Electron spin resonance (ESR) spectra further demonstrated the massive production of ·OH radicals only in the USL group incubated with lactate at pH 6.5 (Figure 2m and Figure S10, Supporting Information), suggesting the potential of using the USL for pH‐selective tumor therapy within the TME.

We then examined the stability of the USL under physiological conditions. The USL catalysts exhibited long‐term stability without apparent morphology and crystallinity changes in phosphate‐buffered saline (PBS, pH 7.4) for 1, 3, and 7 days (Figure S11, Supporting Information). By monitoring the Syr concentrations in buffers, USL was found to exhibit a strong pH‐responsive release profile (Figure S12, Supporting Information), in which Syr was gradually released in weakly acidic media (PBS, pH 6.0) and was barely released under near‐neutral conditions (PBS, pH 7.4). This pH dependence was attributed to the biodegradation capability of ZIF‐8 in an acidic medium, which was evidenced by the significant structural collapse and degradation of the USL through the acidic destruction of ZIF‐8 (Figure S13, Supporting Information). Consequently, this enabled the release of Gd/CeO_2_, LOx, and Syr in acidic media. Therefore, the structural integrity under normal physiological conditions and structural fragility in acidic environments of USL catalysts effectively prevent the premature release of multiple components of USL into normal tissues and selectively deliver various cargoes into tumor sites, suggesting the potential of USL as an intratumorally effective catalyst for pH‐selective tumor therapy via the catalytic depletion of lactate and the production of harmful ·OH.

In addition, USL also exhibited potential for magnetic resonance imaging (MRI) because of the presence of Gd in catalysts, which showed a dose‐dependent increase in brightness under a T1‐weighted MRI (3.0 T, Figure S14a, Supporting Information).^[^ ^48^ ^]^ Subsequently, in vivo MRI images of USL‐injected tumor‐bearing mice demonstrated a significant enhancement in the liver tumor area (Figure S14b, Supporting Information), suggesting the potential of USL as a contrast agent for tumor diagnosis.

### 2.3 In Vitro USL‐Catalyzed Lactate Oxidation for Intracellular Lactate Metabolism and Immune Response

Encouraged by the catalytic performance of USL, its antitumor activity was explored using in vitro cellular experiments. Initially, the engulfment of USL in vitro was evaluated by co‐incubating cells (Hepa1‐6 tumor cells and AML 12 normal cells) with rhodamine‐labeled USL. The uptake of USL by Hepa1‐6 cells was significantly higher than that by AML 12 cells (Figure S15, Supporting Information). Importantly, the lack of apparent cytotoxic effects of USL on normal liver cells demonstrated a high level of biosafety (Figure S16, Supporting Information) due to the structural stability of USL under physiological conditions (Figure S11, Supporting Information).

**Figure 3:**
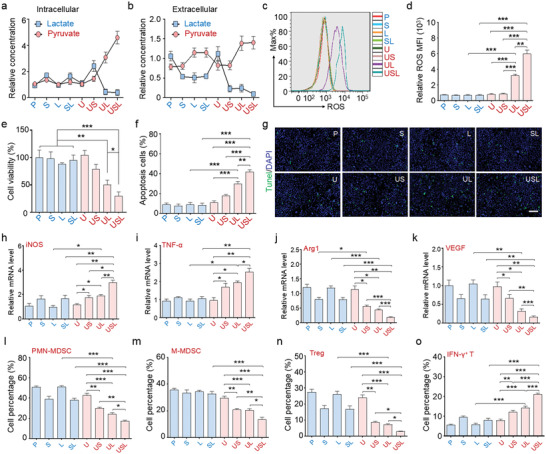
In vitro catalytic performance and antitumor effect of USL‐enabled catalytic lactate oxidation. a,b) Tumor cells were incubated with different treatments for 24 h, and the concentrations of lactate or pyruvate were detected in the cell lysate (intracellular) and supernatant (extracellular). c,d) ROS generation by tumor cells administered different treatments was determined by fluorescence‐activated cell sorting (FACS). e) Relative proliferation of HCC cells after incubating the cells with different treatments. f) Quantitative analysis of apoptosis rates in HCC cells after different treatments. g) TUNEL immunofluorescence staining of cellular apoptosis in different groups. Scale bar = 100 µm. h–k) Stimulated bone marrow‐derived macrophages (BMDMs) were incubated with supernatant from tumor cells administered different treatments for 24 h, and the expression of polarization markers was detected. l–o) The supernatant from treated tumor cells was added to MDSC or naïve T cell culture medium to evaluate the effects on l,m) MDSC generation and n,o) T cell differentiation (*n* = 5). Bars represent the mean ± SEM; *, *p* < 0.05; **, *p* < 0.01; ***, *p* < 0.001.

Based on the above observations, Hepa1‐6 tumor cells were cultured in PBS (P), free Syr (S), free Lox (L), Syr combined with LOx (SL), U, US, UL, or USL. Intra‐ or extracellular lactate and pyruvate concentrations were monitored (**Figure** **3**a,b). The S group showed decreased extracellular lactate levels through inhibition of lactate transport. The administration of L and SL catalyzed the conversion of extracellular rather than intracellular lactate into pyruvate because of their poor cellular uptake efficiency. UL overcame the disadvantages of Lox by catalyzing both intracellular and extracellular lactate.

With the loading of Gd/CeO_2_ only inside ZIF‐8, the nanocarrier exhibited intrinsic inertness for lactate oxidation but facilitated cellular uptake of Syr and Lox. US exhibited a more effective lactate export limitation, and UL overcame the disadvantages of Lox by catalyzing both intracellular and extracellular lactate. Most importantly, the USL catalysts significantly repressed lactate transport and presented the highest capability for the catalytic consumption of both intracellular and extracellular lactate (Figure 3a,b).

Considering that lactate oxidation by Lox could generate a large amount of H_2_O_2_, and Gd‐doped CeO_2_ presented peroxidase‐like activity to convert H_2_O_2_ into toxic ·OH, the catalytic production of ROS was examined with different treatments. The results indicated that the UL and USL catalysts generated excessive intracellular ROS (Figure 3c,d), further facilitating apoptosis and inhibiting tumor cell proliferation (Figure 3e–g and Figure S17–S19, Supporting Information). Syr‐enabled intracellular lactate accumulation provided sufficient reactants for the tandem catalytic processes of lactate oxidation and ROS production driven by LOx and Gd/CeO_2_, respectively. Therefore, USL delivered the highest ability to induce apoptosis (nearly up to 43%) and showed the strongest antitumor capability. In addition, we detected mitochondrial damage after different treatments, indicating that USL clearly damaged the mitochondria of the tumor cells (Figure S20, Supporting Information). Pyruvate, as the product of lactate oxidation, did not influence the proliferation and apoptosis of tumor cells, indicating that the function of the USL catalysts was independent of the oxidation product of pyruvate (Figure S21, Supporting Information).

Lactate metabolism involves immune cell development, including macrophage plasticity, MDSC generation, and T cell activation.^[^ ^10^, ^11^, ^12^ ^]^ Herein, USL participates in immune regulation via both intracellular and extracellular catalytic lactate oxidation. Macrophages have two distinct phenotypes. M1‐polarized macrophages upregulate pro‐inflammatory cytokines and play antitumor roles, whereas M2‐polarized macrophages express anti‐inflammatory genes and promote tumor development.^[^ ^49^, ^50^ ^]^ Among the various treatments, the supernatant of the USL‐treated tumor cells significantly promoted M1 macrophage marker expression while dramatically repressed M2‐associated molecules (Figure 3h–k and Figure S22, Supporting Information). The phosphorylation of STAT6, required for M2 macrophage polarization and pro‐tumor function, was decreased in macrophages incubated with the supernatant of USL‐treated tumor cells (Figure S23, Supporting Information). Additionally, in comparison with other treatments, USL‐triggered tumor cells delivered the best ability to reduce MDSC generation (Figure 3l,m, and Figure S24, Supporting Information).

The catalytic oxidation of tumor cell lactate also regulates T cell differentiation. Comparatively, when stimulated by USL, the supernatant of tumor cells reduced naïve T cell differentiation into regulatory T cells (Tregs) and accelerated cytotoxic T cell development (Figure 3n,o and Figures S25 and S26, Supporting Information). These data strongly indicate that USL significantly alleviated tumor cell growth and regulated the immune response in vitro through catalytic lactate oxidation and reprogrammed metabolism, showing its great potential for tumor therapy.

### 2.4 In Vivo USL‐Catalyzed Lactate Oxidation for Tumor Therapy

**Figure 4:**
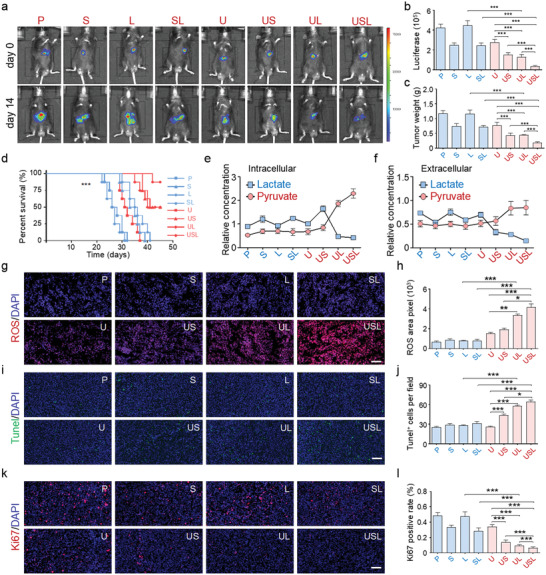
USL repressed tumor progression in vivo. a–c) Orthotopic HCC tumor‐bearing mice were treated by injection of different drugs or biocatalysts. Tumor growth was assessed by in vivo imaging and tumor weight measurements (*n* = 5). d) The survival curve of tumor‐bearing mice undergoing different treatments (*n* = 5). e,f) The concentrations of lactate or pyruvate were detected in the lysate and supernatant of tumor tissues from mice administered different treatments (*n* = 5). g,h) ROS generation in tumors from mice administered different treatments (*n* = 5). i,j) TUNEL staining was performed to evaluate apoptosis in tumors from mice administered different treatments (*n* = 5). k,l) Ki67 staining was performed to assess the proliferation of tumor cells in mice administered different treatments (*n* = 5). Scale bar = 100 µm. Bars represent the mean ± SEM; *, *p* < 0.05; **, *p* < 0.01; ***, *p* < 0.001.

After validating our catalytic lactate oxidation strategy in vitro, in vivo investigations based on an orthotopic hepatocellular carcinoma (HCC) model were performed through the intraperitoneal injection of various drugs and catalysts. During the treatments, the USL showed no obvious lesions or morphological changes in the main organs (Figures S27–S29, Supporting Information). Considering that the potential immunogenicity would limit the application of USL, we injected the catalysts into the periphery of normal mice. The antibody content or cytokine production in the serum was not significantly different, indicating negligible immunogenicity of USL (Figure S30, Supporting Information).^[^ ^51^ ^]^ In addition, the tissue distributions of USL suggested a high tumor affinity of catalysts and its rapid excretion from normal organs through the urine and feces (Figure S31, Supporting Information). These results demonstrated its highly benign in vivo biocompatibility. After two weeks of treatment, tumor growth was quantitatively compared using in vivo imaging and tumor weight measurements. Although S, SL, U, US, and UL attenuated tumor growth, USL displayed the most significant tumor‐inhibitory capability and prolonged the survival time of tumor‐bearing mice (**Figure** **4**a–d). Although LOx catalyzes the conversion of lactate into pyruvate, the complex in vivo conditions limit the application of free LOx. The use of ZIF‐8 improves the stability, loading efficiency, and release. Next, intratumoral catalytic lactate oxidation was examined by monitoring the variations of lactate and pyruvate in both the intracellular and extracellular environments of the digested tumors (details in Methods). Similar to the in vitro results (Figure 3a,b), the USL catalysts exhibited the best catalytic activity for intratumoral lactate oxidation because of the inhibited lactate transportation, active LOx, and efficient cellular uptake of the catalysts in the TME (Figure 4e,f), and did not influence the weakly acidic TME (Figure S32, Supporting Information).

Immunofluorescence staining of tumor tissues suggested that the groups (U, US, UL, and USL) enhanced intratumoral ROS production due to the peroxidase‐like activity of Gd/CeO_2_ (Figure 4g,h). Moreover, due to intratumoral lactate accumulation mediated by Syr and lactate oxidation catalyzed by LOx, USL triggered the most efficient ·OH production (Figure 4g,h). Further immunofluorescence staining demonstrated that the ·OH generated by nanocatalysts induced apoptosis (Figure 4i,j) and inhibited the proliferation (Figure 4k,l) of tumor cells. In particular, USL presented the most effective antitumor function (Figure 4i–l). These results indicate that USL represses HCC progression by catalyzing lactate production and triggering ROS cytotoxicity.

### 2.5 Remodeled Local Immunity via Intratumorally USL‐Catalyzed Lactate Oxidization

**Figure 5:**
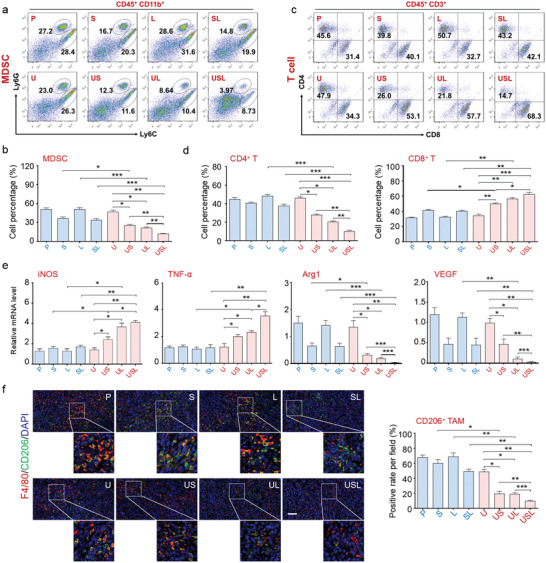
USL switched the immune response from a pro‐tumor phenotype to an antitumor phenotype in vivo. The generation of a,b) MDSC and c,d) T cell subpopulations in tumors from mice administered different treatments was analyzed by FACS, and the percentages were compared (*n* = 5). e) The TAMs in tumors from mice administered different treatments were sorted, and the expression of polarization markers was detected (*n* = 5). f) Immunofluorescence co‐staining for F4/80 and CD206 was carried out to evaluate polarization (*n* = 5). Scale bar = 100 µm. Bars represent the mean ± SEM; *, *p* < 0.05; **, *p* < 0.01; ***, *p* < 0.001.

Accumulated evidence indicates that lactate is involved in tumor immunosuppression.^[^ ^8^, ^10^, ^11^, ^12^ ^]^ The in vitro experiments also demonstrated that USL regulates the immune response via lactate elimination (Figure 3). Therefore, immune cell development in tumors was determined by fluorescence‐activated cell sorting (FACS) in the different groups. USL, owing to its strong activity for lactate oxidation, sharply decreased the number of MDSCs (**Figure** **5**a,b). However, different catalysts did not affect the total T cell proliferation (Figure S33, Supporting Information) but modulated T cell fate determination and Th response type. USL also promoted CD8^+^ cytotoxic T cell differentiation and suppressed CD4^+^ helper T cell development (Figure 5c,d), which induced immune‐mediated killing and decreased immune escape in the TME. Furthermore, although the total (tumor‐associated macrophages, TAMs) number was not regulated by the USL (Figure S34, Supporting Information), the functional plasticity and polarization phenotypes of the TAMs were significantly altered. CD11b^+^F4/80^+^ TAMs were sorted and cultured for functional analysis, which indicated that USL promoted expression of M1 polarization markers and repressed that of M2 polarization markers (Figure 5e). ELISA of the supernatants suggested that USL improved the inflammatory response by facilitating the expression of proinflammatory cytokines (IL‐12 and IL‐6) and decreasing the levels of anti‐inflammatory cytokines (IL‐10 and TGF‐*β*) (Figure S35, Supporting Information). CD206, a critical pro‐tumor marker in macrophages for predicting poor tumor prognosis, was also significantly suppressed by USL (Figure 5f). Taken together, the in vivo results strongly demonstrated the USL‐enabled catalytic oxidation of lactate, remodeled the TME, and promoted antitumor immunity. This enhanced intratumoral immune response by USL effectively represses tumor progression.

### 2.6 Activated Systemic Immune Response via Intratumorally USL‐Catalyzed Lactate Oxidization

**Figure 6:**
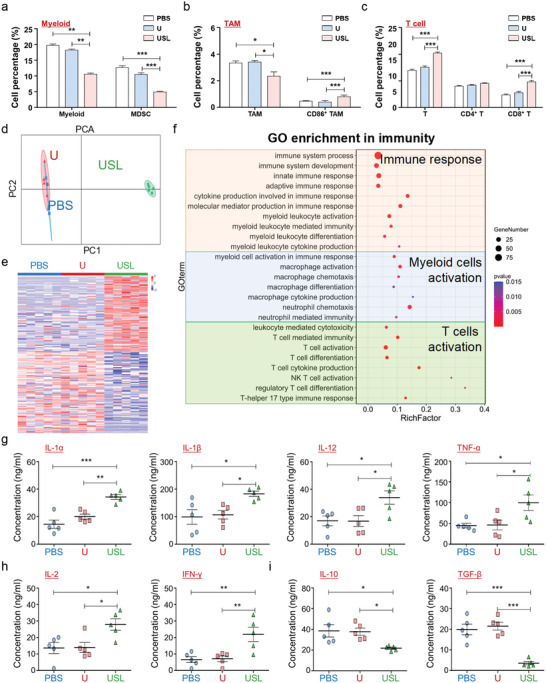
Influence of intratumoral lactate oxidation by the USL catalysts on the systemic immune response through the regulation of paracrine profiles. a–c) The immune response in the spleen was determined by flow cytometry (*n* = 5). d) PCA of gene expression in tumors from mice treated with different therapies (*n* = 5). e) The differentially expressed genes are displayed in a heat map. f) The cluster analysis results are presented based on the dependence on immunoregulatory pathways. g–i) The expression levels of the top distinguished cytokines were validated using ELISA (*n* = 5). Bars represent the mean ± SEM; *, *p* < 0.05; **, *p* < 0.01; ***, *p* < 0.001.

As demonstrated above, USL catalytically improves the local immunity in the TME. However, cancer is a systemic disease that affects the immune system as a whole. Immunoregulation for cancer therapy requires the assessment of the systemic immune landscape beyond the TME.^[^ ^13^, ^15^ ^]^ During tumor development, a large number of immune cells derived from lymphoid organs are continuously recruited into the TME. Many populations of immune cells are re‐educated to develop a pro‐tumor phenotype before penetrating the tumor tissues via metabolites and cytokines in the peripheral blood. Therefore, remodeling of systemic immunity is necessary for tumor therapy. Immune cells were detected in the spleen, indicating that USL inhibited MDSC generation (**Figure** **6**a and Figure S36a, Supporting Information) and promoted M1 macrophage (CD86^+^) differentiation (Figure 6b and Figure S36b, Supporting Information). Moreover, CD8^+^ cytotoxic T cells in the spleen were elevated by USL (Figure 6c and Figure S36c, Supporting Information). Similarly, the immune cells in the peripheral blood were also restored after USL therapy as it decreased MDSC and Tregs and increased CD8^+^ cytotoxic T cells (Figure S37, Supporting Information).

The activation of systemic immunity is inseparable from stimulation of peripheral factors. We then detected the secreted proteins in the serum of tumor‐bearing mice treated with PBS, U, or USL. The secretory proteome of the USL group was distinct from those of the PBS and U groups (Figure 6d). Large immunity‐associated pathways, including macrophage polarization, neutrophil activation, and T cell response, were enriched in the USL group (Figure S38, Supporting Information). Furthermore, the differentially expressed genes (DEGs) in USL‐treated mice were mainly cytokines and chemokines (Figure 6e and Figure S39, Supporting Information) and were related to the regulation of myeloid and lymphoid cell development (Figure 6f). In addition, the expression levels of the representative cytokines were validated by ELISA, which indicated that USL triggered accelerated M1‐polarized macrophage (Figure 6g) and cytotoxic T cell stimulation (Figure 6h), and repressed Treg differentiation (Figure 6i). The remodeling of systemic immunity is the immunological basis for the improvement of the TME, thereby providing a prerequisite for strengthening localized antitumor immunity. The above data demonstrate that USL‐enabled catalytic oxidation of lactate reformed the paracrine pathway in the peripheral serum, which was critical for systemic immunity remodeling.

### 2.7 Effect of In Vivo Catalytic Lactate Oxidation on Tumor Metabolism

**Figure 7:**
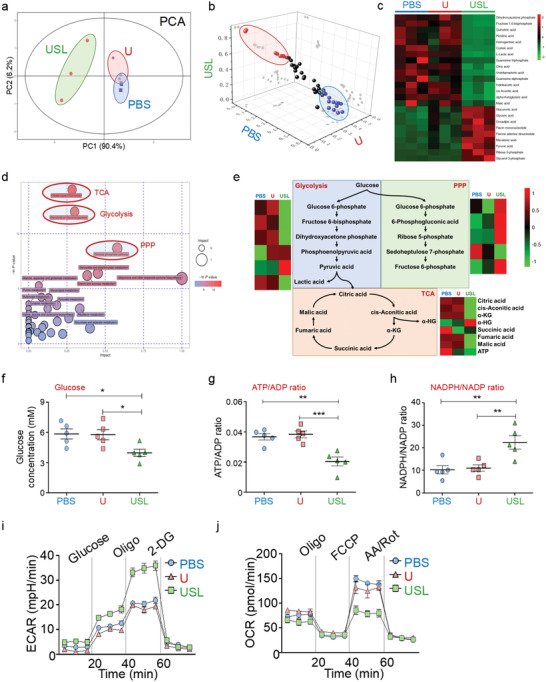
Tumor glycometabolism evolution induced by lactate oxidation catalyzed by various catalysts. a) PCA of the metabolome of tumors from mice treated with various therapies. b,c) The metabolites of different groups are displayed in a 3D diagram and heat map. d) The cluster analysis results are presented according to the metabolic pathways and functions. e) The flow diagram of central carbon metabolism. The critical metabolites are marked in different colors according to the relative concentrations. f–h) Glucose uptake, ATP/ADP ratios, and NADPH/NADP^+^ ratios were determined in tumor tissues from mice undergoing different therapies. i,j) Glycolytic activity (ECAR) and oxygen consumption (OCR) of tumor‐associated macrophages from different groups were detected using a Seahorse analyzer (*n* = 5). Bars represent the mean ± SEM; *, *p* < 0.05; **, *p* < 0.01; ***, *p* < 0.001.

During tumor progression, the metabolism disorder and nutrition seizing of tumors results in a suppressive immune response and attenuates immunotherapy through the paracrine pathway. Lactate, acting as a metabolite and metabolic driver, plays an important role in the regulation of metabolism. It is reasonable to speculate that USL‐enabled lactate catalysis improves both local and systemic immunity via reprogrammed tumor metabolism. Therefore, we performed metabolomic analysis of tumors treated with PBS, U, or USL. Principal component analysis (PCA) indicated that the metabolites from USL‐treated tumors were clearly different from those of the other groups (**Figure** **7**a). Fifteen downregulated and nine upregulated metabolites were observed in USL‐treated mice (Figure 7b,c), which were primarily enriched in glycolysis, the tricarboxylic acid (TCA) cycle, and the pentose phosphate (PPP) pathway (Figure 7d). Furthermore, as revealed from the flow diagram of central carbon metabolism, USL dramatically inhibited glycolysis and the TCA cycle but promoted the PPP pathway (Figure 7e). The metabolic regulation of USL was dependent on lactate catalysis, without influencing the expression of glycol‐metabolism enzymes or transporters (Figure S40, Supporting Information). As demonstrated above, USL effectively consumed intratumoral lactate and greatly enhanced intratumoral pyruvate accumulation, which in turn inhibited glycol‐metabolism (Figure 7e) and glucose uptake (Figure 7f) through a negative feedback. However, excessive intratumoral ROS generation by USL (Figure 4g,h) resulted in mitochondrial dysfunction and attenuated TCA cycle activity (Figure 7e), leading to a reduction in ATP synthesis (Figure 7g) and NADPH consumption (Figure 7h). Interestingly, USL‐induced ROS‐damaged DNA and the DNA repair process required a large amount of ribose (supported by the PPP pathway). Therefore, the USL catalysts promoted the PPP pathway, which competitively reduced glycolysis and the TCA cycle, and further induced starvation therapy for antitumor activity through the inhibition of glycometabolism (Figure 7e).

USL‐mediated metabolic reprogramming causes glucose accumulation and metabolic waste deprivation in the TME, which intrinsically regulate the development of immune cells. M1 macrophages preferentially utilize glycolysis, whereas M2 macrophages primarily use mitochondrial oxidative phosphorylation for energy generation.^[^ ^7^ ^]^ After USL treatment, TAMs increased glycolysis (Figure 7i) and reduced oxidative phosphorylation (Figure 7j), suggesting a switch towards M1 polarization. The above data demonstrated that USL‐catalytic intratumoral lactate oxidation reprogrammed the metabolism of tumor cells and evoked antitumor immunity in the TME. In this study, we clarified the mechanisms of USL‐enabled lactate oxidation on antitumor immunity regulation. USL can elicit robust local and systemic immune responses through catalytic metabolic reprogramming, which effectively represses tumor growth. In addition, elucidating the effects of tumor catalytic therapy on energy metabolism and immunomodulation in the TME is crucial for improving the efficacy of tumor treatment, which also contributes to a more comprehensive understanding of tumor metabolism and immune reprogramming for tumor therapy.

## 3 Discussion

**Figure 8:**
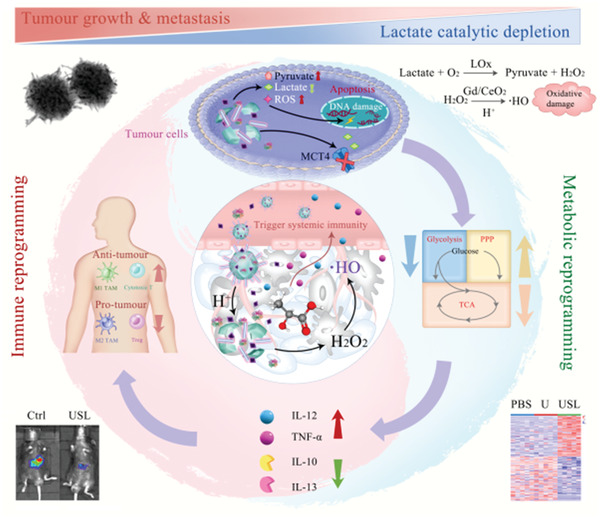
Schematic illustration of the USL‐enabled intratumoral lactate oxidation, sequential metabolic reprogramming, and immunity enhancement, as well as the in situ catalytic production of ·OH for tumor therapy.

Overall, USL shows a high affinity for tumor tissues and releases active components only in the slightly acidic environment of tumors, which satisfies therapeutic targeting and presents potential biosafety in tumor therapy, as illustrated in **Figure** **8**. First, in the dedicatedly designed USL, acid‐soluble ZIF‐8, as a cargo delivery with a spiky morphology, enriches the catalysts in tumors and then releases all components into the TME. Syr limits lactate transport, LOx enables lactate‐to‐pyruvate oxidation and H_2_O_2_ production, and Gd/CeO_2_ with peroxidase‐like activity produces toxic ·OH in the weakly acidic TME, while barely exhibiting activity under neutral environments and exerting protective functions in normal organs. Second, USL‐catalyzed lactate oxidation reprograms metabolism in the TME by inhibiting glycolysis and the TCA cycle. Third, metabolic reprogramming remodels and restores both local and systemic antitumor immunity. USL‐catalyzed metabolic alteration in the TME triggers TAM M1‐polarization, depresses MDSC differentiation, and evokes a Th1 response. Meanwhile, the decrease in lactate in serum induces changes in the cytokine profile in peripheral blood, which influences immune cell development in lymphoid organs and ameliorates the systemic immune response. Lastly, USL‐enabled catalytic intratumoral ·OH production and lactate oxidation‐evoked immune therapy synergistically achieve catalytic therapy and immunotherapy of tumors. Both pathways intensified the antitumor ability of USL.

Our study and other groups have demonstrated the antitumor function of LOx induced by lactate. However, previous studies have mainly focused on the ROS generation ability and tumor cell cytotoxicity of LOx and ignored its influence on the TME. In fact, lactate catalysis or depletion modulates metabolic alterations in tumor cells, inducing the reprogramming of metabolite profiles and immune responses in the TME. Lactate also directly influences the development of multiple immune cells, such as macrophages, MDSC, and NK cells. Considering the complex regulation of lactate metabolism and immunity, we attempted to comprehensively elucidate the functions and mechanisms of tumor repression by lactate catalysis.

In this study, we provide a preliminary exploration of this catalytic metabolic reprogramming strategy and a systematic understanding of the catalytic conversion of intratumoral metabolites, reprogrammed metabolism, and evoked local and systemic immunity for tumor therapy. Systemic immunity remodeling could intercept the resources of pro‐tumor immune cells and maintain antitumor immune memory, indicating that this strategy might present long‐term therapeutic efficacy and repress tumor recurrence. This design strategy is also promising in the therapeutics of other metabolism‐related diseases, such as Alzheimer's disease and atherosclerosis. We anticipate that catalytic metabolic reprogramming will open up a plethora of possibilities in the fields of disease treatment and biomedicine.

## 4 Experimental Section

### Synthesis and Modification of Gd/CeO_2_

All Gd/CeO_2_ catalysts were prepared by a modified hydrothermal method.^[^ ^43^ ^]^ Briefly, 0.09 mmol of Ce(NO_3_)_3_ (Sigma‐Aldrich, USA) was dissolved in 30 mL of deionized water in polytetrafluoroethylene vessels. After the complete dissolution of cerium nitrate, 0.005 mmol of Gd(NO_3_)_3_ (Sigma‐Aldrich, USA), dissolved in 10 mL of deionized water, was added to the Ce(NO_3_)_3_ solution. Under magnetic stirring, ammonium hydroxide (0.1 m, Sigma‐Aldrich, USA) was added dropwise to complete the precipitation (final pH = 10) and stirred at 160 °C for 24 h. The products were collected by centrifugation, washed with deionized water, and redispersed in water to form a 1.0 mg mL^−1^ solution. Then, 20 mL of an aqueous solution of polyvinylpyrrolidone (0.5 g, PVP 55 000 Da, Sigma‐Aldrich, USA) was added to 10 mL of the Gd/CeO_2_ solution and stirred for 24 h. The PVP‐modified Gd/CeO_2_ was finally separated by centrifugation at 12000 rpm for 30 min, washed with pure water three times, and dispersed in pure water for future use.

### Synthesis of USL

Typically, 10 mL of 2‐methyl imidazole (25 × 10^−3^ m, Sigma‐Aldrich) and 10 mL of Zn (NO_3_)_2_·6H_2_O (25 × 10^−3^ m, Sigma‐Aldrich) were incubated at room temperature overnight. Gd/CeO_2_ was added at the beginning of the reaction, whereas LOx (Yuanye Bio‐Technology, China) and syrosingopine (MedChemExpress, USA) were added 5 min after the reaction was initiated. The product was then separated by centrifugation, washed three times with pure water, and dispersed in pure water for future use.

### Characterization

High‐resolution TEM images and elemental mapping images were obtained using a field‐emission transmission electron microscope operated at 200 kV (JEOL JEM‐F200). The XRD pattern was recorded using a powder diffractometer (Shimadzu, Model 6000) in the 2*θ* range of 5–90°. X‐ray photoelectron spectroscopy (XPS) spectra were collected using a K‐Alpha Thermo Electron Model. Dynamic light scattering and zeta potential measurements (Nano ZS90 Zetasizer, Malvern) and Fourier transform infrared spectroscopy (FTIR, NICOLET 6700) were used to characterize the functional groups and stability of the nanostructures. Nitrogen adsorption/desorption isotherms were measured at 77 K using a BSD‐PM2 (BeiShiDe, China) gas adsorption analyzer after the sample was first degassed at 150 °C overnight. Specific surface areas were determined using the Brunauer–Emmet–Teller method, while total pore volumes were determined using the adsorption branch of the N_2_ isotherm at *p*/*p*_0_ = 0.99 (multiple points). Between 40–60 mg of the material was used for each measurement. Tissue distributions of the USL were analyzed using inductively coupled plasma mass spectrometry. ROS were identified using an ESR spectrometer (Bruker, USA).

### Syr Release

USL (30 mg) was dispersed in 5 mL of PBS buffer at pH 6.0 and 7.4, and then incubated at 37 °C with gentle shaking. Then, 0.2 mL of buffer solution was removed periodically to detect Syr. The Syr dose was measured by HPLC.

### Peroxidase‐Like Activity

Gd/Ce@ZIF and Gd/Ce&LOx@ZIF were dispersed in water to obtain a final concentration of 1 mg mL^−1^ and then sonicated for 30 s before use. The Gd/Ce@ZIF and Gd/Ce&LOx@ZIF suspensions (20 µL) were added to 975 µL of acetate buffer (20 × 10^−3^ m, pH 6.0). Next, 5 µL of 300 × 10^−3^ m tetramethylbenzidine was added to the above mixture. The mixture was then reacted at 25 °C for 10 min and absorbance recorded immediately on a UV‐Vis spectrophotometer at 652 nm (Perkin Elmer).

### Bioactivity and Stability of LOx@ZIF

The bioactivity and stability of 10 µg LOx and 10 µg immobilized LOx in ZIF were compared using lactate as a reactant in PBS (50 × 10^−3^ m, pH 7.0) at room temperature for 30 min. After the reaction, the LOx activity was evaluated based on the amount of hydrogen peroxide in the product. The concentration of hydrogen peroxide was determined using a colorimetric assay performed according to the manufacturer's instructions after PBS dilution for quantitative analysis (Quantitative Peroxide Assay Kit, 23280, Thermo).

### In vitro and In Vivo MRI Tests

The in vitro MRI experiments of the USL with different concentrations were performed using an MRI scanner (3.0 T, uMR 780, United Imaging). USL with Gd concentrations ranging from 2–22 × 10^−6^ m was dispersed in water. The in vitro T1‐weighed MRI was performed with the following scan parameters: slice thickness = 1.0 mm, TE = 277.2 ms, TR 2000 ms; bandwidth, 520 Hz Px^−1^. In vivo MRI was performed using 3.0 T magnetic resonance imaging (uMR 780, United Imaging). When the tumor size approached ≈100 mm^3^, tumor‐bearing mice were administrated USL via intravenous injection (5 mg kg^−1^), and the MRI was obtained at the time point of 15 min after the injection. Parameter settings: TR = 2000, TE = 277.2.

### Mice and Tumor‐Bearing Mouse Model

C57BL/6 mice were maintained in a specific pathogen‐free facility. All animal experiments were approved by the Animal Experiment Administration Committee of the Fourth Military Medical University to ensure ethical and humane treatment of animals (KY20183341‐1). For orthotopic HCC models, the Hepa1‐6 cells were infected with lentivirus expressing firefly luciferase (GeneChem, Shanghai, China) to construct stable tumor cell line. The stable tumor cells could emit fluorescence in the presence of luciferin both in vivo and in vitro detection. The mice were anesthetized by intraperitoneal (i.p) injection of 0.6% pentobarbital sodium (10 µL g^−1^; Sigma‐Aldrich, St. Louis, MO). Then the tumor cells (5 × 10^6^) were suspended in 30 µL Matrigel (Sigma‐Aldrich) and inoculated into liver parenchyma of left lobe for in situ HCC model. One week later, different drugs were administered to the tumor‐bearing mice via intravenous injection for therapy. Mice were sacrificed 3 weeks after inoculation, and hepatic tumor growth was monitored using an in vivo imaging system (IVIS) (Xenogen, Perkin‐Elmer, Fremont, CA, USA). In addition, part of the tumor was preserved in 4% paraformaldehyde for pathological analysis, and the rest of the tumor was minced and digested into a single cell suspension by 40 min incubation with type V collagenase and DNase I for further analysis. For some experiments, tumor‐associated macrophages were sorted by FACS using an AriaIII flow cytometer (BD Immunocytometry Systems) from a single‐cell suspension of tumors.

### Cell Culture and Cell Differentiation

Nucleated cells in the bone marrow (BM) of C57BL/6 mice were cultured in Dulbecco's modified Eagle's medium (DMEM) that contained 10% fetal calf serum (FCS) and 2 × 10^−3^ m L‐glutamine (Gibco, Waltham, MA). For bone marrow‐derived macrophage (BMDM) stimulation, M‐CSF (25 ng mL^−1^) (SinoBio, Beijing, China) was added to the cultured cells for 7 d. In some experiments, LPS (10 ng mL^−1^) and IFN‐*γ* (20 ng mL^−1^) were used to trigger M1 polarization, while IL‐4 (20 ng mL^−1^) was used to induce M2 polarization. For MDSC stimulation, GM‐CSF (25 ng mL^−1^) and IL‐6 (25 ng mL^−1^) (SinoBio, Beijing, China) were added to the cultured cells for 4 d. The HCC cell lines Hepa1‐6, H22, and Hca‐F were obtained from the American Type Culture Collection (ATCC, Manassas, VA) repository in 2017 and cultured in DMEM supplemented with 10% FCS and 2 × 10^−3^ m l ‐glutamine.

### Lactate Production

Prepared cells (5×10^6^ cells) were lysed with 500 µL of double‐distilled water (DDW). According to the protocol of the lactate concentration determination kit (Jiancheng, Nanjing, China), the working buffer and coloration reagent were mixed, and cell lysate or supernatant was added to the reaction solution and incubated at room temperature (RT) for 10 min. Absorbance was measured at 530 nm using a microplate reader (Sunrise, Tecan Group Ltd., Switzerland).

### Pyruvate Production

The prepared cells (5×10^6^ cells) were lysed with 500 µL of DDW and deproteinized with a 10 K spin column to remove the proteins that consumed pyruvate. The reaction mixture was then added to each well containing the prepared test samples, according to the recommended protocol (AmyJet Scientific, Wuhan, China). The reaction mixtures were incubated for 30 min at RT in the dark and the absorbance at 570 nm was measured using a microplate reader (Sunrise).

### NAD^+^/NADH Ratio Determination

The NAD^+^/NADH ratio was determined using the NAD/NADH‐Glo Assay (Promega, Madison, WI, USA) according to the manufacturer's protocol. MDSC subsets subjected to different treatments were seeded in 96‐well plates with 50 µL culture medium. The plates were then removed from the incubator and equilibrated at RT for 5 min. Then, 50 µL of NAD/NADH‐Glo detection reagent was added to each well. After incubation for 45 min at RT, luminescence signals were recorded using a luminometer (Promega).

### ROS Generation Detection

The cultured cells were collected and stained with 2’,7’‐dichlorofluorescein diacetate (Abcam, Cambridge, MA, USA).^[52]^ After incubation for 30 min at 37 °C, the fluorescence intensity of the ROS signal in different cell subsets was determined using flow cytometry. In some immunofluorescence experiments, the tumor tissue samples were stained with a ROS probe.

### Ce Concentration Detection

To the dried tissues and samples (including urine), aqua regia was added and heated at 80 °C to completely digest the samples. Then, the solutions were diluted with water, and the Ce content was determined by inductively coupled plasma MS (iCAP 7400, Thermo Scientific).

### RNA Extraction and Reverse Transcription‐Polymerase Chain Reaction (RT‐PCR)

Total RNA was extracted from cultured cell samples using TRIzol reagent (Invitrogen) according to the manufacturer's instructions. Complementary DNA was prepared using a reverse transcription kit (Vazyme, Nanjing, China) to detect the expression levels of macrophage polarization markers. Quantitative real‐time PCR was performed using the ChamQ SYBR qPCR Master Mix Kit (Vazyme) and ABI PRISM 7500 Real‐time PCR System in triplicate (Life Technologies, Waltham, MA), with GAPDH as the internal control. The PCR primers used are listed in Supplementary Table S1.

### Liver and kidney function detection

Whole blood samples from the mice that underwent various treatments were centrifuged at 3000 rpm for 15 min at 4 °C. The supernatant was then separated immediately for detection or stored at −80 °C. The working reagent was prepared according to the manufacturer's instructions and incubated with the serum samples. Liver and kidney function indices were determined using an automatic biochemical instrument (Chemray 800, Rayto).

### HE Staining

The sections were dewaxed as per the standard procedure and rinsed with tap water. The stained sections were immersed in hematoxylin solution for 3–5 min and rinsed with tap water. The sections were then treated with hematoxylin differentiation solution and rinsed with tap water. Thereafter, the sections were treated with Hematoxylin Scott Tap Bluing and rinsed with tap water. Finally, the stained sections were treated with eosin dye for 5 min after treatment with 85% ethanol and 95% ethanol. The dehydrated sections were observed by microscopic inspection and images were acquired and analyzed.

### Immunofluorescence Staining

The tumor tissues from mice were fixed with 4% paraformaldehyde, followed by antigen retrieval. After blocking with 5% BSA, sections were incubated with different antibodies and stained with 4’,6‐diamidino‐2‐phenylindole (DAPI). The sections were observed under a laser scanning confocal microscope (FV‐1000, Olympus, Tokyo, Japan). The antibodies used are listed in Table S2.

### Flow Cytometry

The cells were stained with different antibodies, as listed in Table S2. FACS analysis was performed according to routine protocols using a FACS AriaIII flow cytometer (BD Immunocytometry Systems). The data were analyzed using FlowJo vX.0.6 software (FlowJo, LLC, Ashland, OR, USA). Dead cells were excluded by 7‐AAD staining. To validate the engulfment of USL by tumor cells, multispectral imaging FACS was performed to detect rhodamine‐labeled USL in a single cell using ImageStreamX (Merck, Darmstadt, Germany). In some cases, the Annexin V Apoptosis Detection Kit (eBioscience, Waltham, MA) was used to detect tumor cell apoptosis. FITC‐Annexin V was added to a single‐cell suspension and incubated for 15 min at RT, followed by propidium iodide staining. The cells were then washed with 1× annexin‐binding buffer and gently mixed for further analysis by flow cytometry.

### Cell Energy Essay

The TAMs were sorted from PBS‐, U‐, or USL‐treated mice and resuspended in Seahorse XF DMEM base medium. The oxygen consumption rate (OCR) and extracellular acidification rate (ECAR) were measured using a Seahorse XFp extracellular flux analyzer (Agilent Technologies, Santa Clara, CA, USA). Briefly, TAMs were sequentially exposed to oligomycin, trifluoromethoxy carbonylcyanide phenylhydrazone (FCCP), and rotenone/antimycin A for OCR detection. For ECAR detection, TAMs were treated with glucose, oligomycin, and 2‐deoxy‐D‐glucose (2‐DG).

### Enzyme‐Linked Immunosorbent Assay

Stimulated bone marrow‐derived macrophages (BMDMs) were incubated with tumor cells treated with different nanomaterials. In some experiments, TAMs from different groups of tumor‐bearing mice were sorted using FACS for further culturing. Then, the supernatant of BMDMs or TAMs was harvested, and cytokine concentrations were determined using an ELISA kit (eBioscience), according to the manufacturer's instructions. In the immunogenicity detection assay, the total antibody content or cytokine production was determined by ELISA using serum from the treated mice.

### Proliferation Assays

The proliferation of tumor cells was analyzed using the CCK‐8 assay. Tumor cells that underwent different treatments were seeded in 96‐well plates, and cell proliferation was evaluated at 1, 2, 3, and 4 days using CCK reagent buffer. After incubation for 4 h at 37 °C, spectrophotometric absorbance was measured at a wavelength of 450 nm using a microplate reader (Sunrise).

### Colony Formation Assay

The colony formation ability of the tumor cells was determined after the indicated nanodrugs were administered. Prepared cells were seeded at a density of 1000 cells per well in a 6‐well plate and incubated for three weeks until cell colonies had formed. After removing the medium and rinsing with PBS, the colonies were fixed with methanol and stained with a 0.5% crystal violet solution (Sigma) for 30 min at RT. The samples were then washed completely with DDW and examined under a light microscope (Olympus, Tokyo, Japan) to count the number of colonies.

### High‐Pressure Ion Chromatography (HPIC)‐MS/MS Analysis

Tissue samples were added to 500 µL of precooled MeOH/H_2_O (3/1, v/v). They were then homogenized for 4 min at 40 Hz and sonicated for 10 min in an ice water bath. Homogenization and sonication cycles were repeated three times, followed by incubation at −40 °C for 1 h and centrifugation at 12000 rpm and 4 °C for 15 min. The supernatants (400 µL) were collected and dried. Then, 250 µL of water was added to the dried residue to create a reconstituted solution. The reconstituted samples were vortexed before filtration through a filter membrane, and subsequently transferred to inserts in injection vials for HPIC‐MS/MS analysis. HPIC separation was performed using a Thermo Scientific Dionex ICS‐6000 HPIC System (Thermo Scientific) equipped with Dionex IonPac AS11‐HC (2× 250 mm) and AG11‐HC (2 mm×50 mm) columns. An AB SCIEX 6500 QTRAP^+^ triple quadrupole mass spectrometer (AB Sciex) equipped with an electrospray ionization (ESI) interface was used for assay development. The typical ion source parameters were IonSpray voltage = ‐4500 V, temperature = 450 °C, ion source gas 1 = 45 psi, ion source gas 2 = 45 psi, and curtain gas = 30 psi. AB SCIEX Analyst Work Station software (1.6.3 AB SCIEX), MultiQuant 3.0.3 software, and Chromeleon7 were employed for MRM data acquisition and processing.

### Statistical Analysis

The images were imported into Image‐Pro Plus 5.1 software (Media Cybernetics Inc., Bethesda, MA, USA), and the densities of the electrophoretic bands were quantified. The data were analyzed using GraphPad Prism 5 software (version 5.0; GraphPad Software, San Diego, CA). Unpaired student's t‐tests, paired t‐tests, or one‐way ANOVA with Tukey's multiple comparison tests were performed for statistical analysis. The statistical results are expressed as the mean ± SEM. Statistical significance was set at *p* < 0.05.

## Conflict of Interest

The authors declare no conflict of interest.

## Author Contributions

J. Z., Z.T., S. Z., and D.F. contributed equally to this work. L.L., Y.Q., J.Z., and Z.T. conceived and designed the study. J. Z., Z. T., S. Z., D. F., Z. G., L. W., Y. Z., F. X., J. Z., S. M., J. H., and T. J. performed the experiments and analyzed the data. T.J., Y.Q., D.C., and L.L. supervised the study. Y.Q., L.L., J.Z., and Z.T. wrote and edited the manuscript. All authors have discussed the results and contributed to the preparation of the manuscript.

## Supporting information

Supporting Information

## Data Availability

The data that support the findings of this study are available from the corresponding author upon reasonable request.
