# Supplementary material for: Insights into the Effect of Catalytic Intratumoral Lactate Depletion on Metabolic Reprogramming and Immune Activation for Antitumoral Activity
Source: Adv Sci (Weinh). 2022 Dec 7;10(4):2204808. doi: 10.1002/advs.202204808 (PMC9896070; doi:10.1002/advs.202204808)
Supplement: Supplementary file 1 — Supporting Information [file ADVS-10-2204808-s001.pdf]

ADVANCED SCIENCE
Open Access

# Supporting Information

for *Adv. Sci.*, DOI 10.1002/advs.202204808

Insights into the Effect of Catalytic Intratumoral Lactate Depletion on Metabolic Reprogramming and Immune Activation for Antitumoral Activity

*Junlong Zhao, Zhimin Tian*, Shoujie Zhao, Dayun Feng, Zhixiong Guo, Liangzhi Wen, Yejing Zhu, Fenghua Xu, Jun Zhu, Shouzheng Ma, Jie Hu, Tao Jiang, Yongquan Qu*, Dongfeng Chen* and Lei Liu**

Supporting Information

**Insights into the Effect of Catalytic Intratumoral Lactate Depletion on Metabolic Reprogramming and Immune Activation for Antitumoral Activity**

*Junlong Zhao, Zhimin Tian,* Shoujie Zhao, Dayun Feng, Zhixiong Guo, Liangzhi Wen, Yejing Zhu, Fenghua Xu, Jun Zhu, Shouzheng Ma, Jie Hu, Tao Jiang, Yongquan Qu,* Dongfeng Chen,* and Lei Liu**

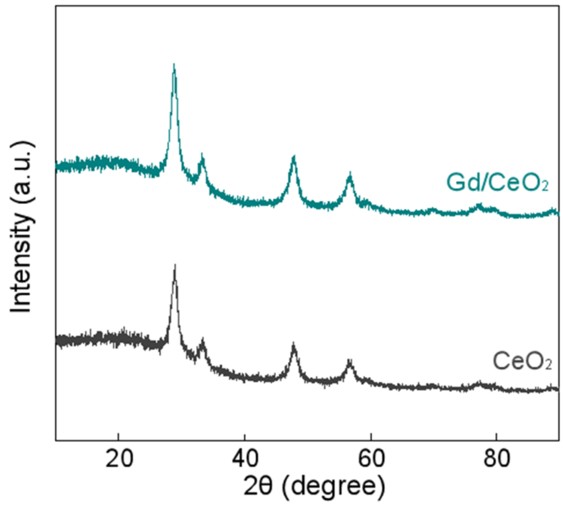


**Figure S1.** X-ray diffraction (XRD) patterns of $CeO_2$ and Gd/$CeO_2$.

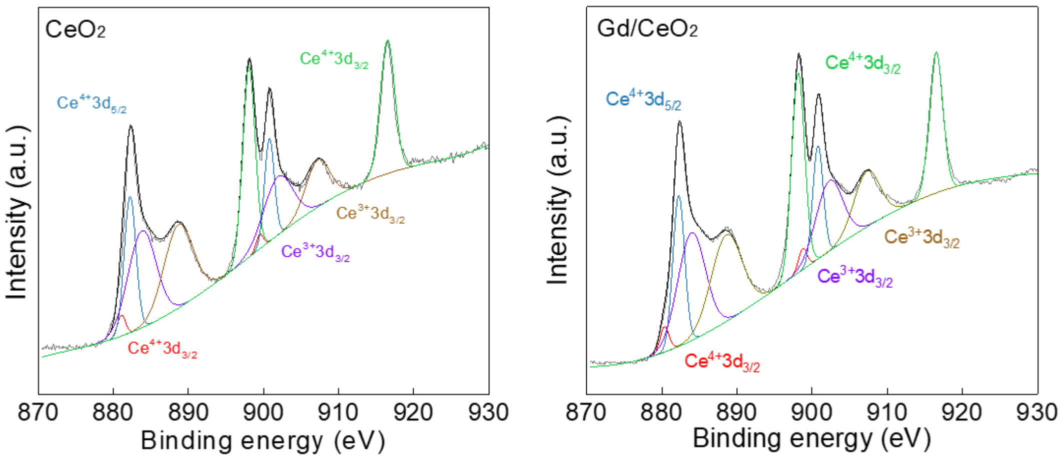


**Figure S2.** X-ray photoelectron spectroscopy survey spectra of $CeO_2$ and Gd/$CeO_2$.

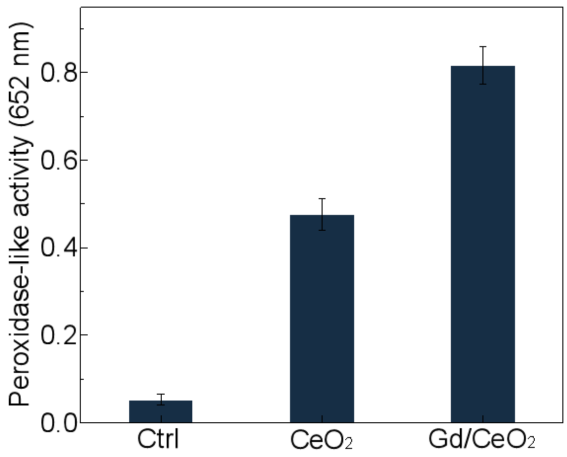


**Figure S3.** Peroxidase-like activities of $CeO_2$ and Gd/$CeO_2$ at 25 °C for 10 min (acetate buffer, 20 mM, pH 6.0). The peroxidase-like activities of $CeO_2$ and Gd/$CeO_2$ were investigated by using TMB as the substrate and were quantificationally indicated by the absorbance value of the oxidation state of TMB at 652 nm.

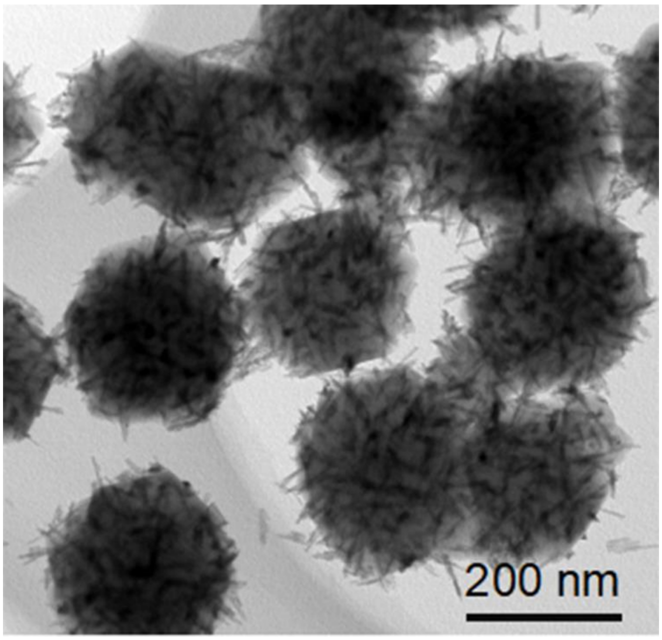


**Figure S4.** Transmission electron microscopy image of USL.

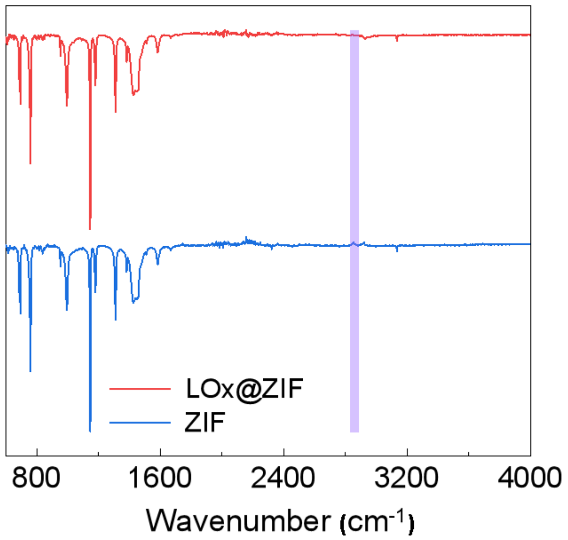


**Figure S5.** Fourier transform infrared (FT-IR) spectroscopy spectra for Gd/$CeO_2$@ZIF and Gd/$CeO_2$&LOX@ZIF. The FT-IR spectrum of Gd/$CeO_2$@ZIF displayed characteristic C-N stretching at 1146 $cm^{-1}$ and ring stretching at 1310-1460 $cm^{-1}$, which further confirms the formation of ZIF-8 in the catalysts. Additionally, the characterized vibrations of LOx molecules, which showed characteristic LOx bands, including methylene at 2930 $cm^{-1}$, further demonstrated the successful immobilization of LOx on Gd/$CeO_2$&LOX@ZIF.

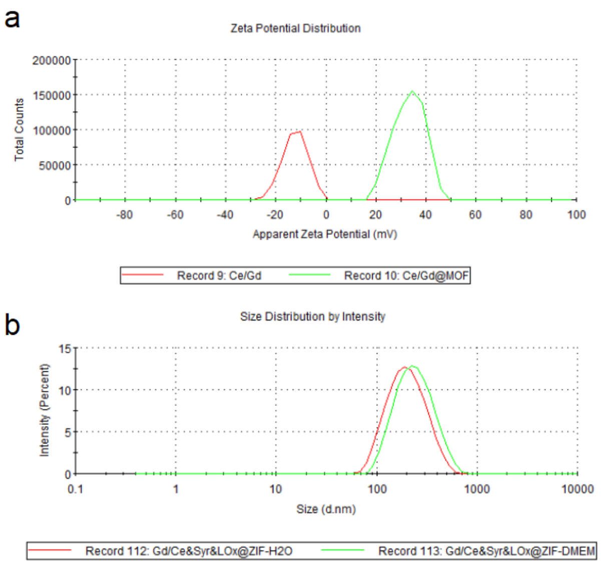


**Figure S6.** a) Zeta potential results for Gd/$CeO_2$ and Gd/$CeO_2$@ZIF. b) Dynamic light scattering results for USL in different solutions ($H_2O$ and Dulbecco's modified Eagle's medium).

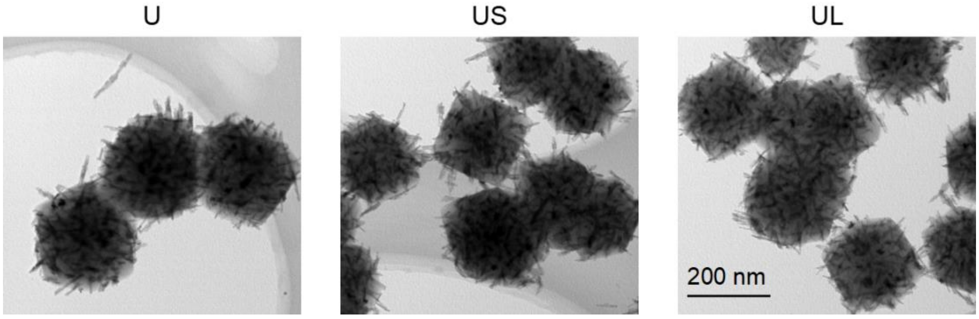


**Figure S7.** Transmission electron microscopy image of U, US and UL.

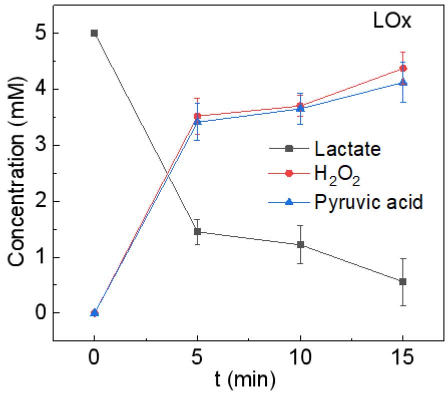


**Figure S8.** Comparison of the catalytic oxidation of lactate by LOx. The enzymatic activity of LOx was evaluated by tracing the decomposition of lactate and the formation of pyruvic acid and $H_2O_2$. The reaction is lactate + $O_2$ → pyruvic acid + $H_2O_2$. The concentrations of LOx were controlled at 200 μg/mL. The reaction conditions were 37 °C for 10 min in PBS (pH 6.0).

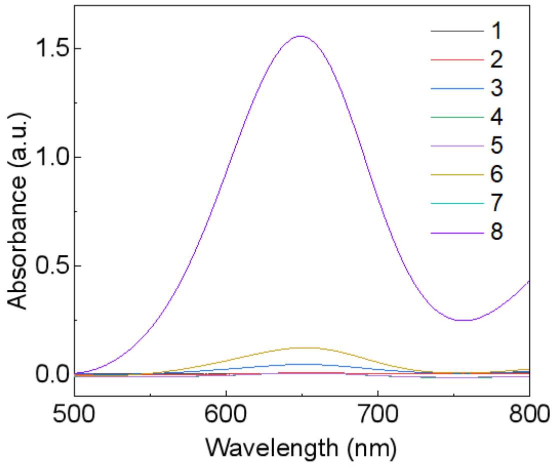


**Figure S9.** Peroxidase-like activities of different catalysts under different conditions. Absorbance at 652 nm of TMB reaction solutions catalysed by different catalysts at 37 °C for 10 min (acetate buffer, 20 mM, pH 6.0). 1: TMB; 2: Lactate + TMB; 3: LOx + TMB; 4: Lactate + LOx + TMB; 5: US + TMB; 6: US + Lactate + TMB; 7: USL +TMB and 8: USL + Lactate +TMB.

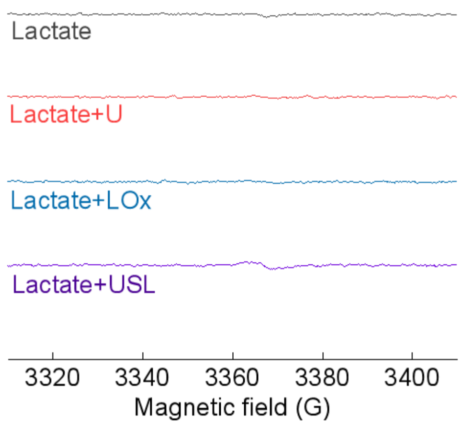


**Figure S10.** Electron spin resonance spectra of lactate, lactate + U, lactate + LOx and lactate + USL in NaAc buffer (pH = 7.4) by using 5-tert-butoxycarbonyl-5-methyl-1-pyrroline-N-oxide as the •OH trapping agent.

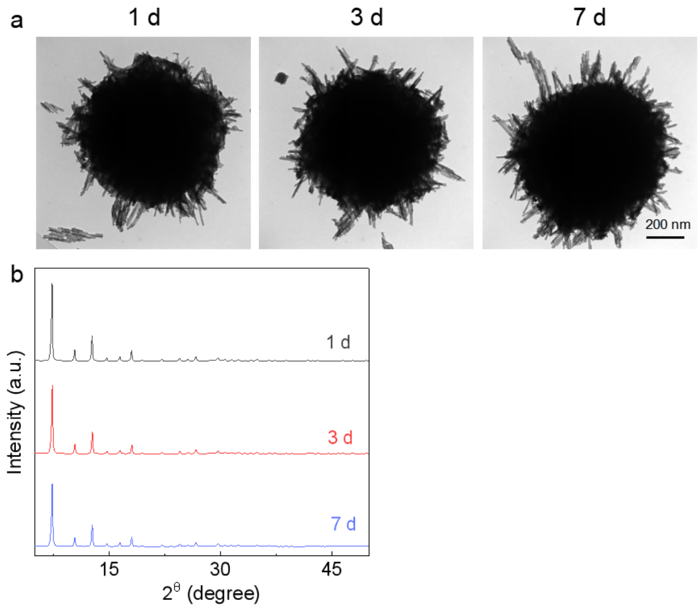


**Figure S11.** TEM images and XRD patterns of USL during incubation in phosphate buffer (pH 7.4) for 1-7 day.

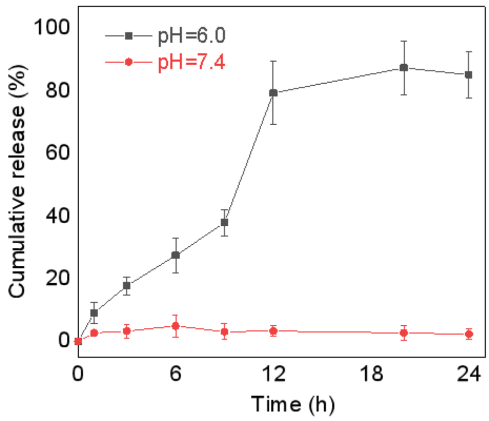


**Figure S12.** Cumulative release profiles of syrosingopine from USL in phosphate buffer (pH 6.0 and 7.4) at 37 °C.

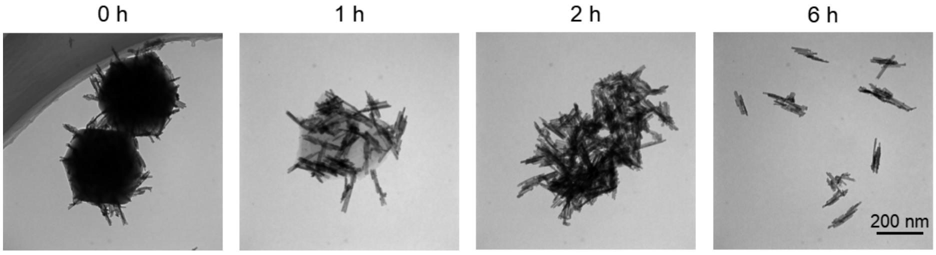


**Figure S13.** TEM images of USL incubated in acidic medium for various durations.

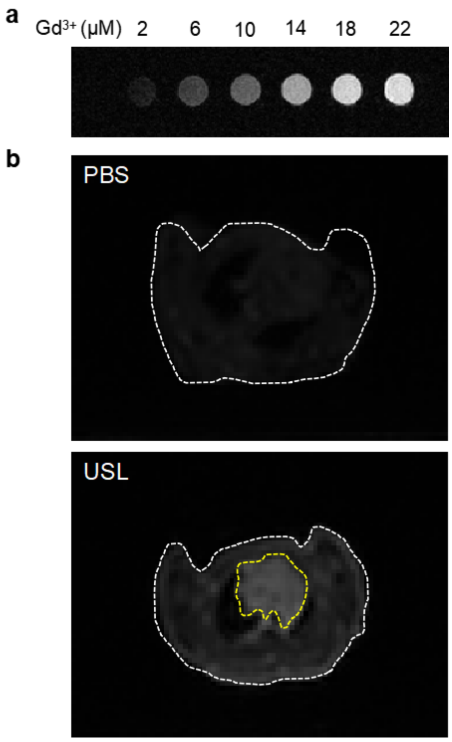


**Figure S14.** a) Magnetic resonance imaging (3.0 T) images of USL at various $Gd^{3+}$ concentrations. b) In vivo magnetic resonance imaging images of tumor-bearing mice after injection of PBS or USL (5 mg/kg).

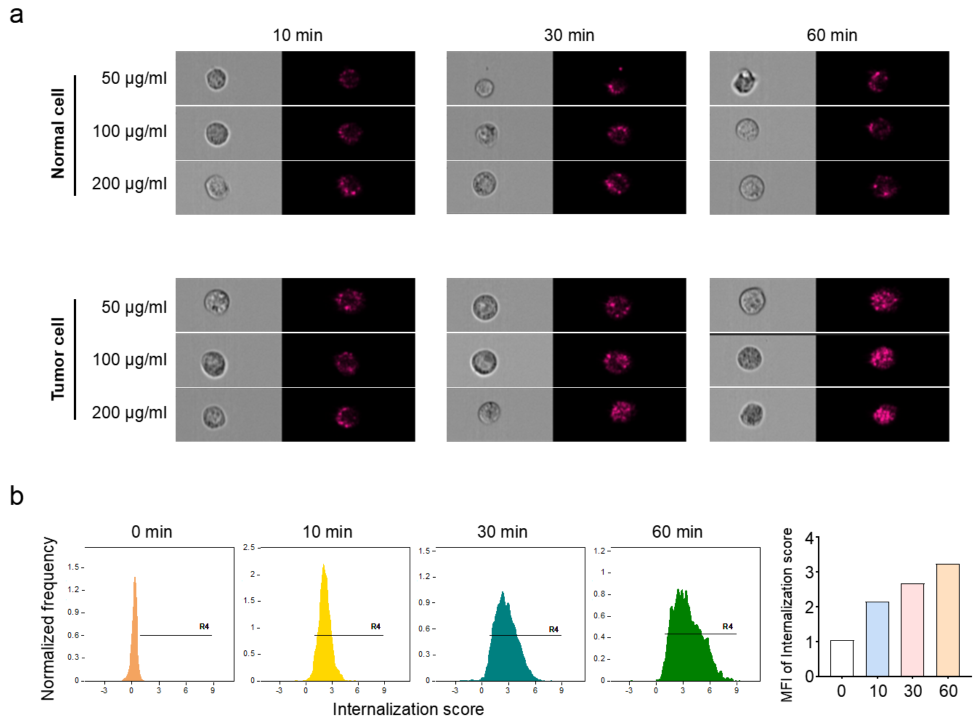


**Figure S15.** a) Rhodamine-labeled USL was co-incubated with Hepa1-6 tumor cells or AML12 normal cells and the engulfment of USL was evaluated by imagine fluorescence-activated cell sorting (FACS). b) The engulfment of USL was quantitied analyzed in different time.

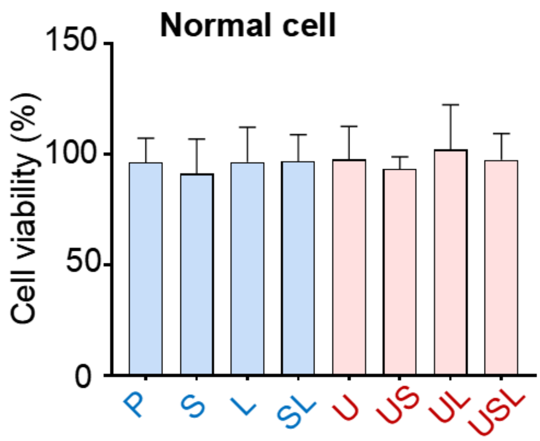


**Figure S16.** The proliferation of AML12 normal liver cells with different treatment by CCK8 assay to evaluate the bio-safety in vitro.

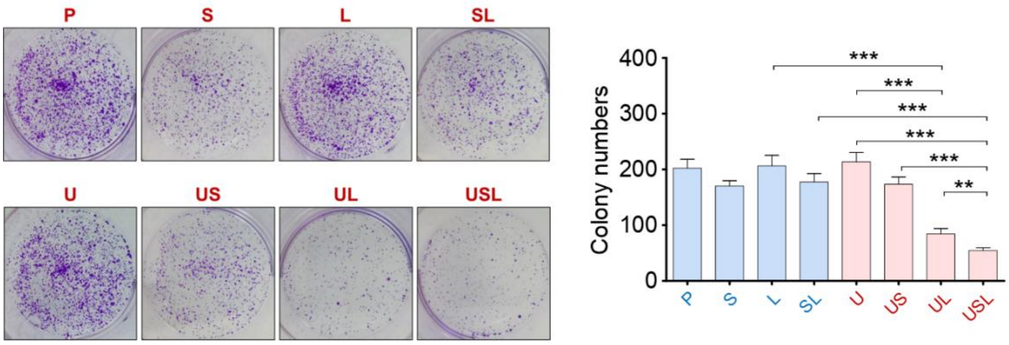


**Figure S17.** USL repressed tumor cell proliferation was detected by colony formation assays. Bars, mean ± SEM; **, $P < 0.01$; ***, $P < 0.001$.

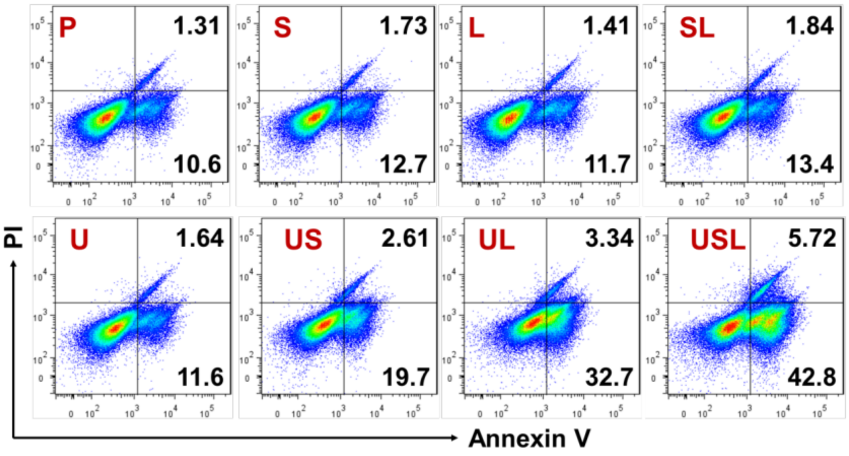


**Figure S18.** Tumor cells were incubated with different treatments, and apoptosis was determined by FACS.

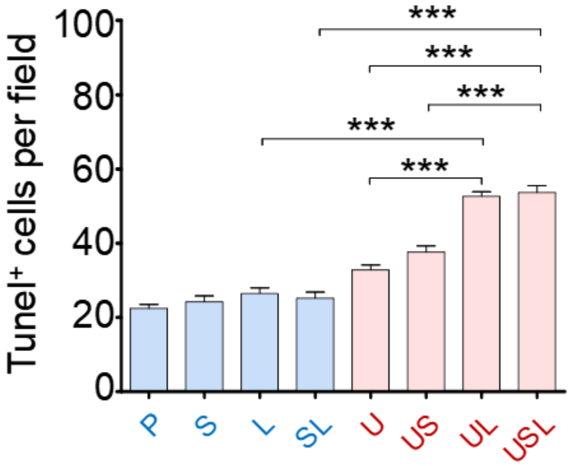


**Figure S19.** Tumor cells were incubated with different treatments, and apoptosis was determined by TUNEL staining. Bars, mean ± SEM; ***, P < 0.001.

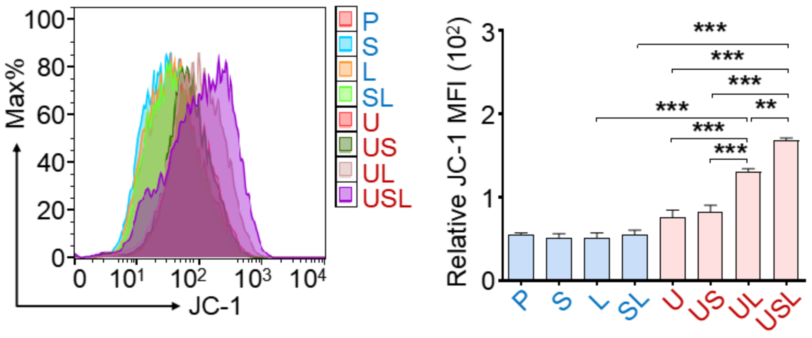


**Figure S20.** JC-1 staining of tumor cells with different treatments. Bars, mean ± SEM; **, P < 0.01; ***, P < 0.001.

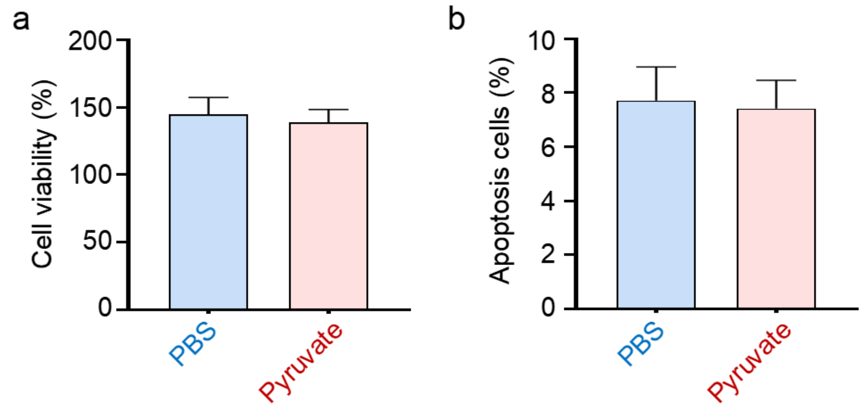


**Figure S21.** The influence of pyruvate on tumor cells. Bars, mean ± SEM.

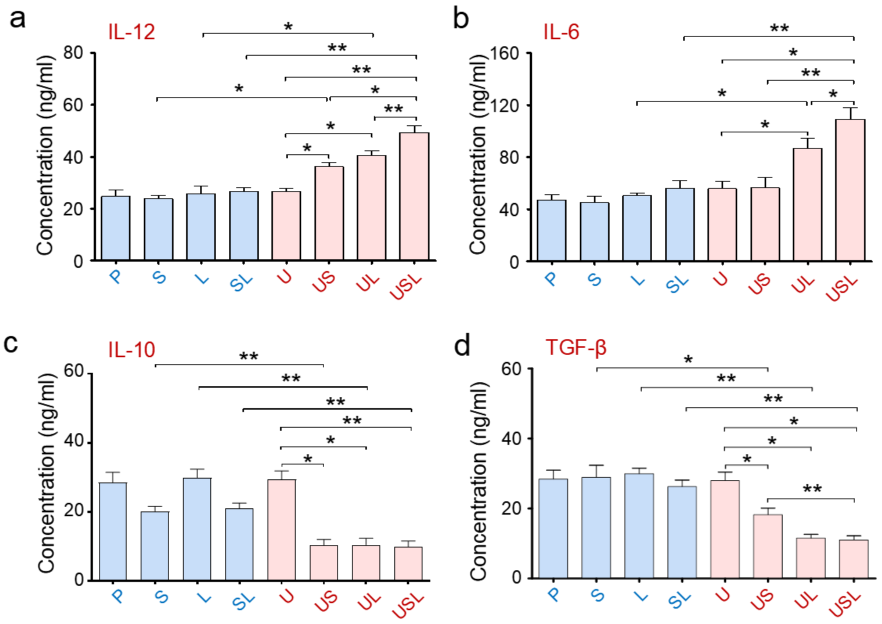


**Figure S22.** USL-treated tumor cells increased the pro-inflammatory cytokine production of macrophages. Macrophages were cocultured with treated tumor cells, and cytokine production was determined by ELISA (n = 5). Bars, mean ± SEM; *, $P < 0.05$; **, $P < 0.01$.

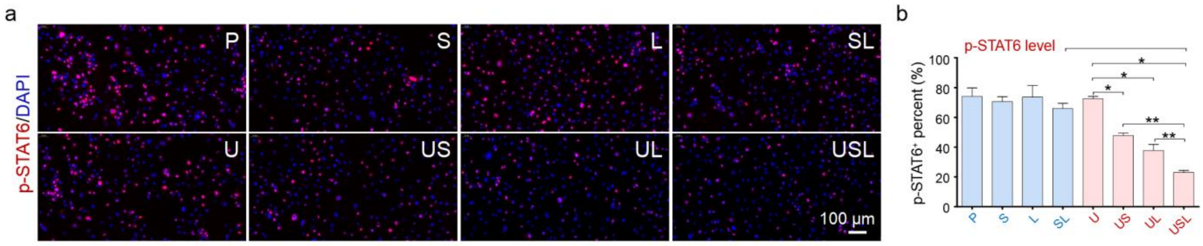


**Figure S23.** USL-treated tumor cells decreased the activation of STAT6 in macrophages. Macrophages were cocultured with treated tumor cells, and the phosphorylation of STAT6 was detected by immunofluorescence staining (n = 5). Bars, mean ± SEM; *, $P < 0.05$; **, $P < 0.01$; ***, $P < 0.001$.

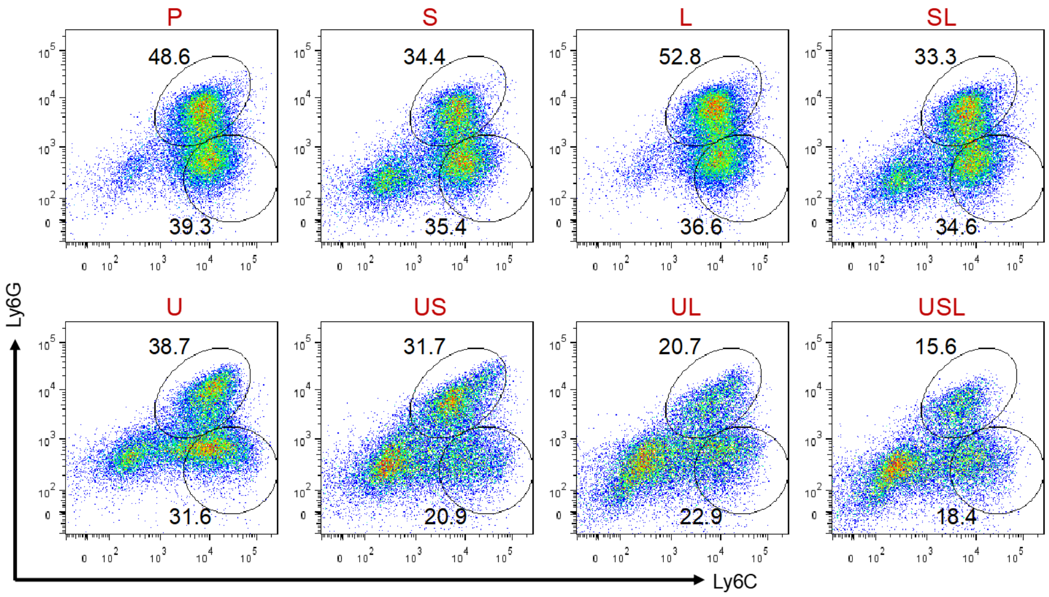


**Figure S24.** USL-treated tumor cells repressed MDSC generation. Myeloid cells isolated from the BM were cocultured with treated tumor cells, GM-CSF and IL-6 for 4 days. Then, MDSC generation was determined by FACS.

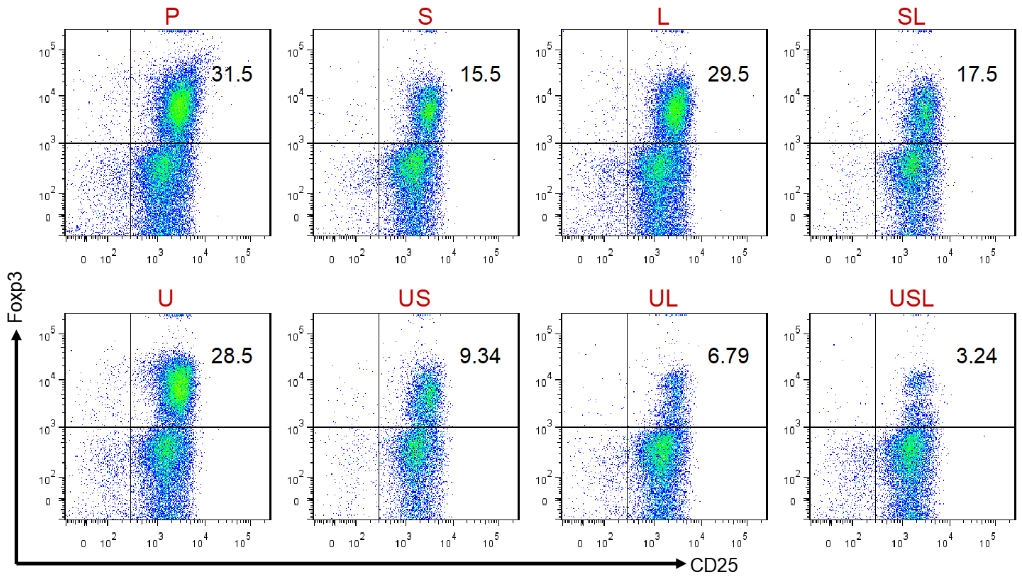


**Figure S25.** USL-treated tumor cells repressed Treg differentiation. Naïve T cells were isolated from the spleen and cocultured with treated tumor cells, IL-2 and TGF-β for 5 days. Then, the differentiation of Tregs was determined by FACS.

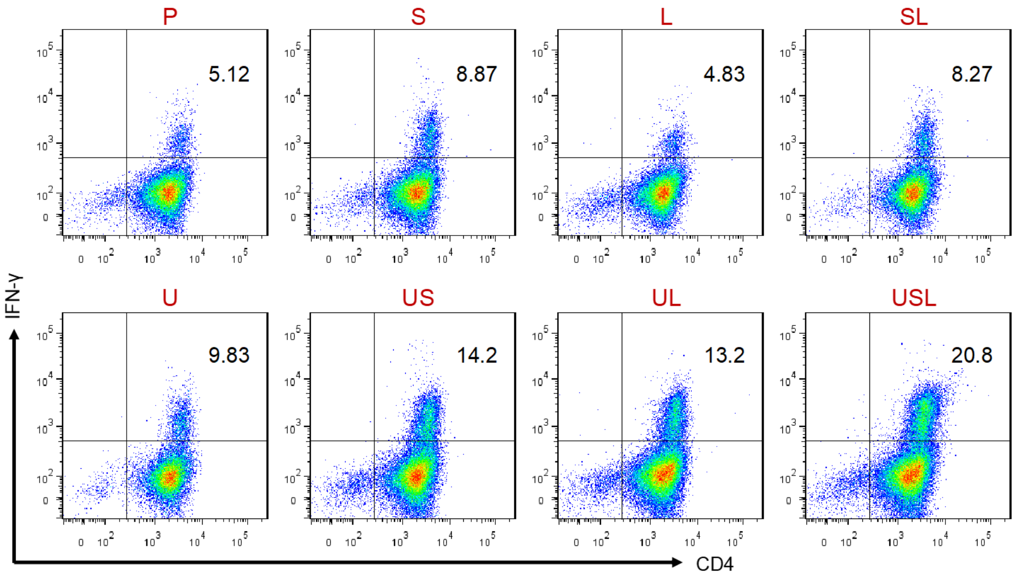


**Figure S26.** USL-treated tumor cells promoted Th1 cell differentiation. Naïve T cells were isolated from the spleen and cocultured with treated tumor cells, IL-2 and IL-12 for 5 days. Then, the differentiation of Th1 cells was determined by FACS.

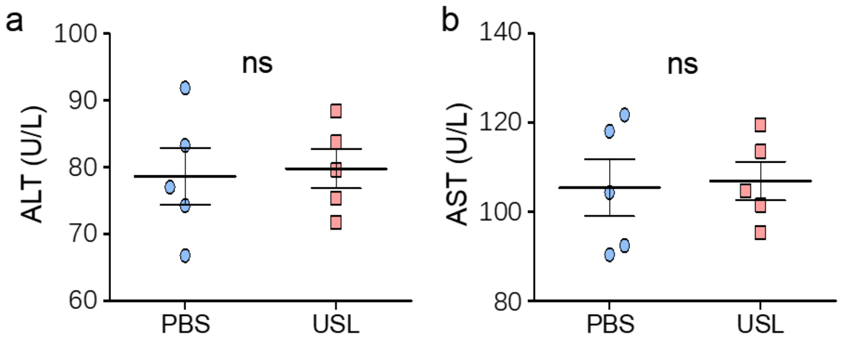


**Figure S27.** USL did not affect the liver function of mice. Serum was isolated from mice injected with USL, and liver function was evaluated. Bars, mean ± SEM; ns, non-sense.

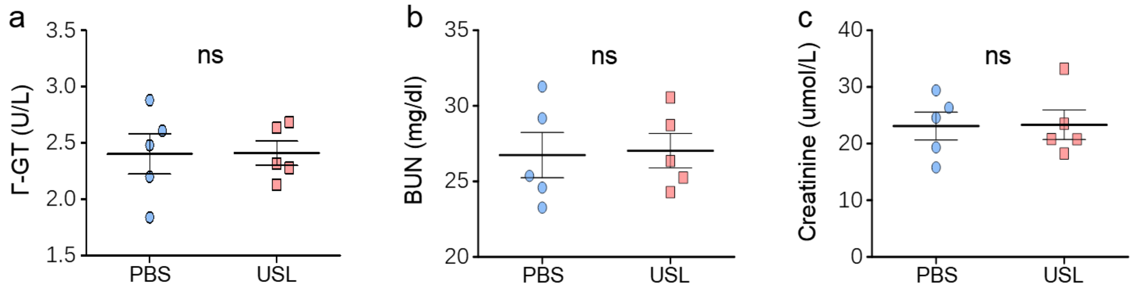


**Figure S28.** USL did not affect the kidney function of mice. Serum was isolated from mice injected with USL, and kidney function was evaluated. Bars, mean ± SEM; ns, non-sense.

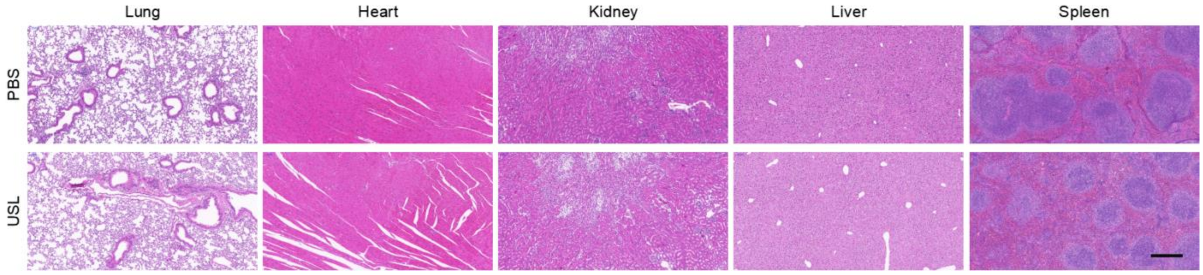


**Figure S29.** USL did not affect the normal morphology of different tissues. USL was injected into normal mice (i.v.), and different tissues were isolated for HE staining. Scale bar = 2 mm.

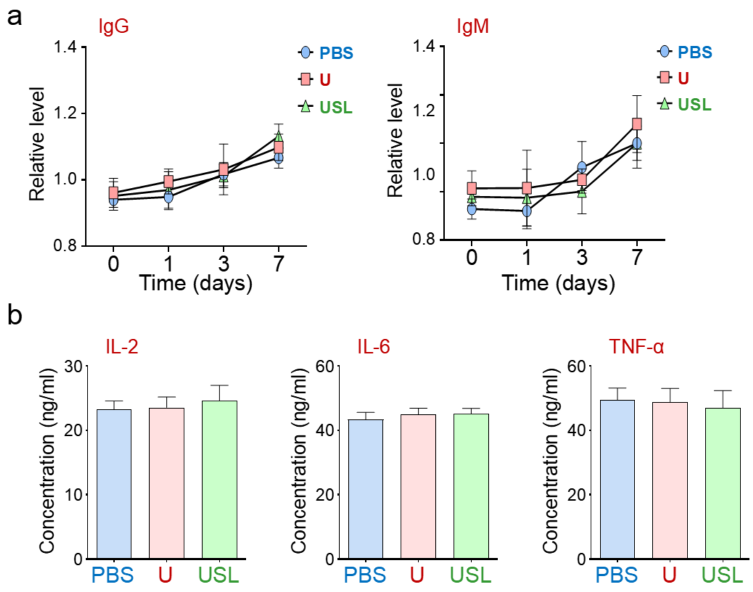


**Figure S30.** The different catalysts were injected into the peripheral blood of normal mice. a) The total IgG and IgM were detected in serum after different time. b) The cytokine production in serum was determined.

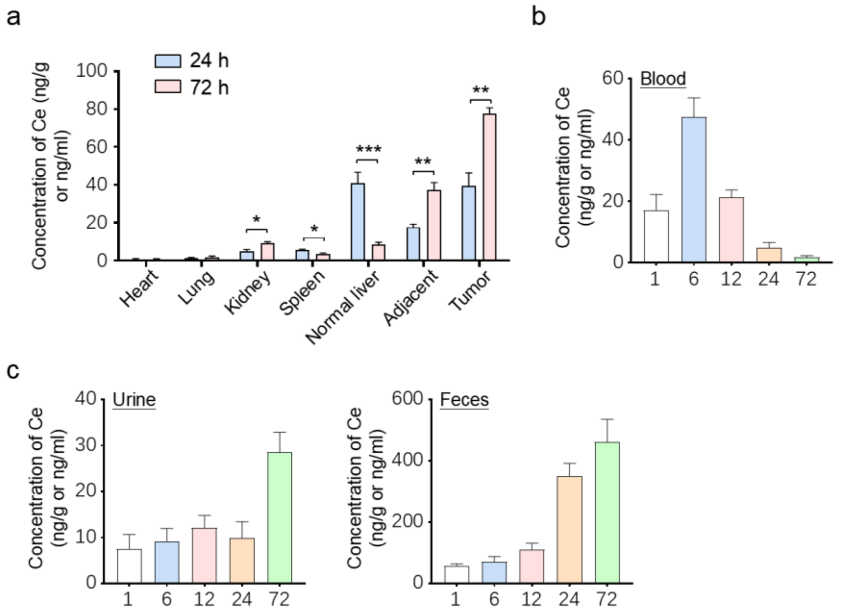


**Figure S31.** USL accumulated in the tumor and liver. USL was injected into normal mice (i.v.), and the consectration of Ce was detected in different tissues a) blood b) urine and feces c) by ICP. Bars, mean ± SEM; *, $P < 0.05$; **, $P < 0.01$; ***, $P < 0.001$.

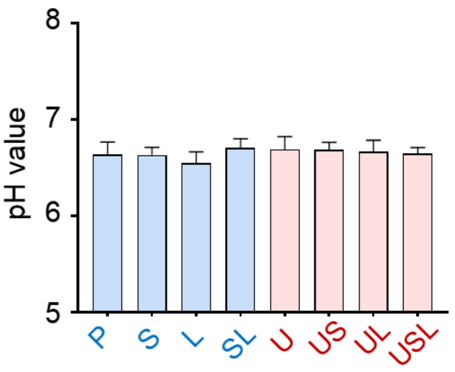


**Figure S32.** The pH value in suspension of tumors from mice in different treatment groups. Bars, mean ± SEM.

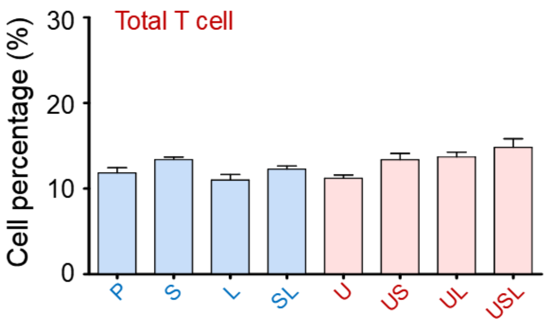


**Figure S33.** USL did not affect the T cell percentage. Tumor-bearing mice were injected with different treatments, and the percentage of T cells was detected by FACS. Bars, mean ± SEM.

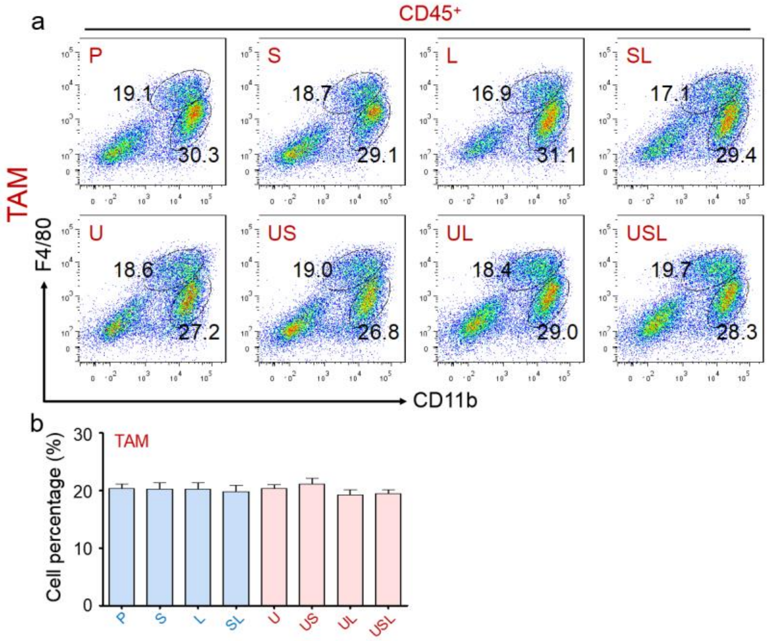


**Figure S34.** USL did not affect the total TAM percentage. Tumor-bearing mice were injected with different treatments, and the percentage of TAMs was detected by FACS. Bars, mean ± SEM.

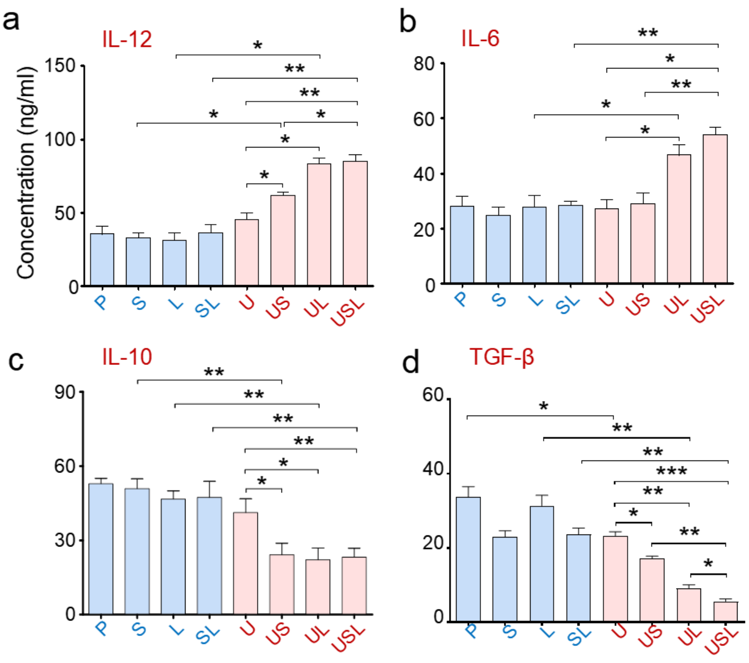


**Figure S35.** USL promoted pro-inflammatory cytokine production in tumor-bearing mice. Tumor-bearing mice were injected with different treatments, and the serum was isolated for cytokine detection by ELISA. Bars, mean ± SEM; *, $P < 0.05$; **, $P < 0.01$; ***, $P < 0.001$.

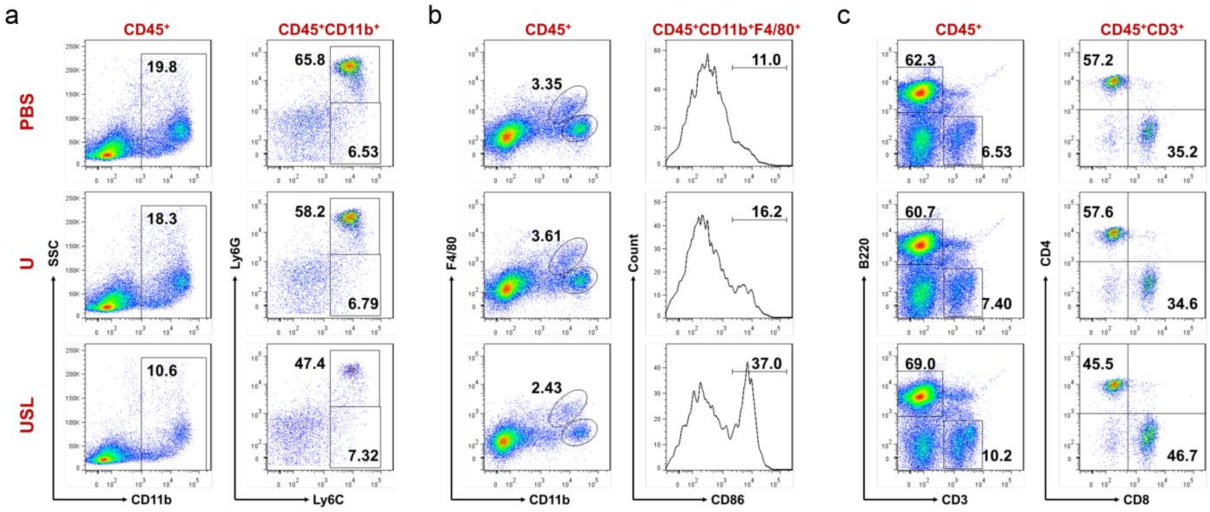


**Figure S36.** The MDSCs generation a) macrophage development b) and T cell differentiation c) in spleen of mice after various treatments were determined by FACS.

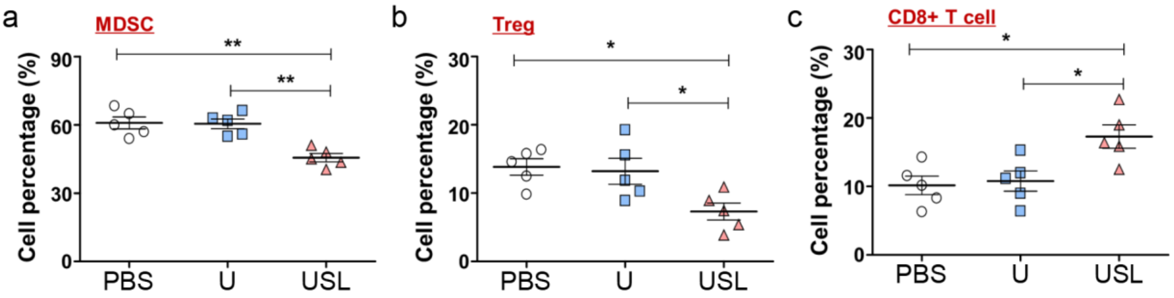


**Figure S37.** USL regulated immune cell development in the peripheral blood of tumor-bearing mice. Tumor-bearing mice were injected with different treatments, and the immune cell subpopulations in the peripheral blood were detected by FACS. Bars, mean ± SEM; *, P < 0.05; **, P < 0.01.

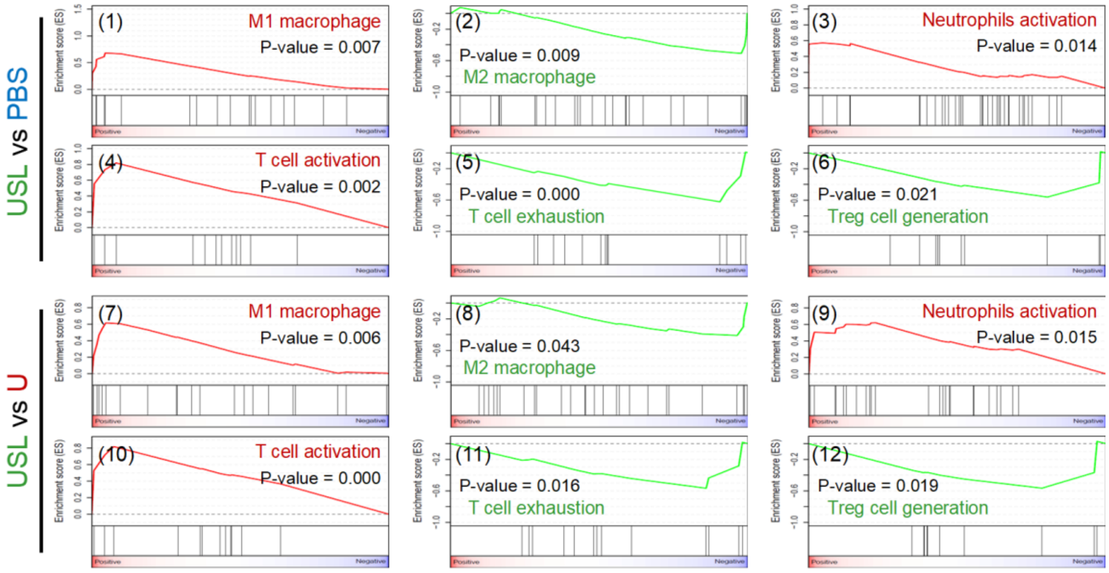


**Figure S38.** The enrichment of differentially expressed genes was investigated by GSEA.

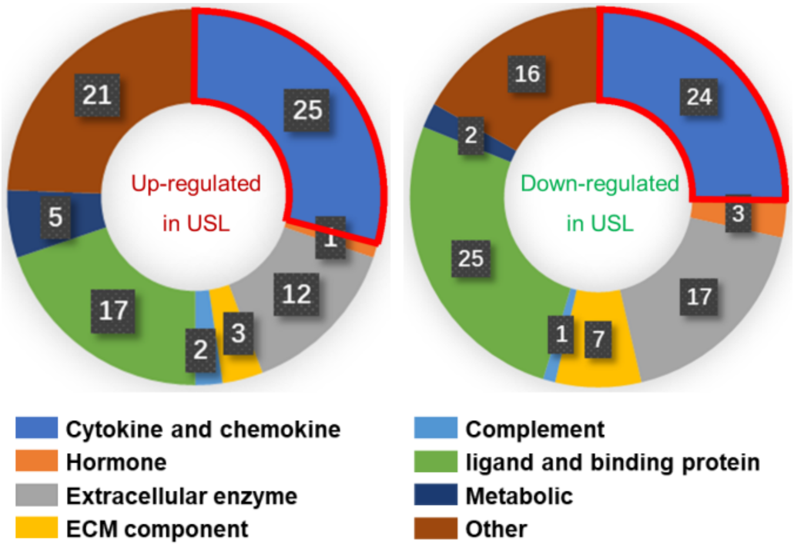


**Figure S39.** The classification of differentially expressed genes is presented.

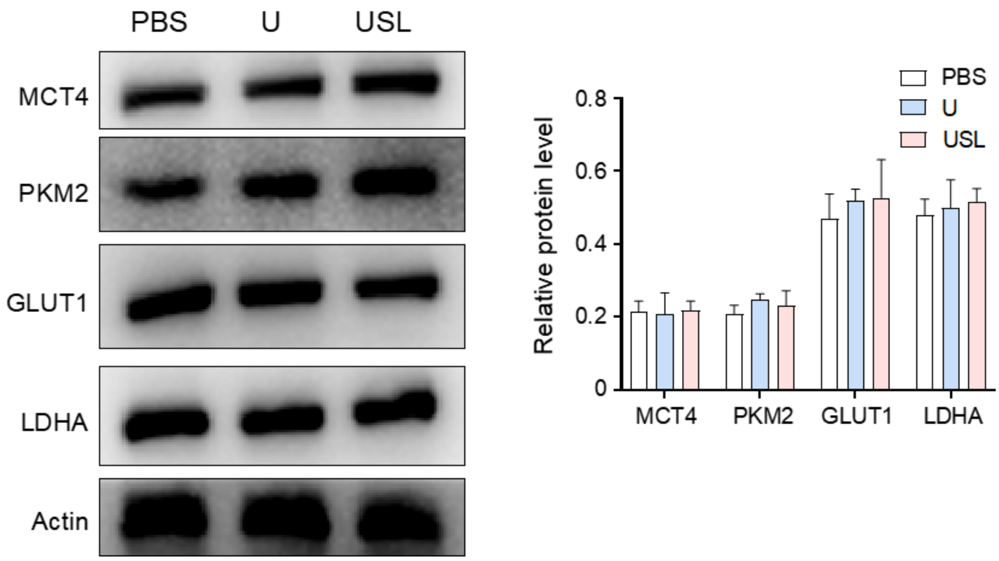


**Figure S40.** The expression of metabolism related enzymes and transporters influenced by USL.

**Table S1.** Primers used in this study.

| Name | Assay | Sequence |
|---|---|---|
| iNOS-F[a)] | RT-PCR | 5'-GCAGAGATTGGAGGCCTTGTG-3' |
| iNOS-R | RT-PCR | 5'-GGGTTGTTGCTGAACTTCCAGTC-3' |
| TNF-α-F | RT-PCR | 5'-CAGGAGGGAGAACAGAAACTCCA-3' |
| TNF-α-R | RT-PCR | 5'-CCTGGTTGGCTGCTTGCTT-3' |
| Arg1-F | RT-PCR | 5'-CTCCAAGCCAAAGTCCTTAGAG-3' |
| Arg1-R | RT-PCR | 5'-AGGAGCTGTCATTAGGGACATC-3' |
| VEGF-F | RT-PCR | 5'-CTGCCGTCCGATTGAGACC-3' |
| VEGF-R | RT-PCR | 5'-CCCCTCCTTGTACCACTGTC-3' |
| Actin-F | RT-PCR | 5'-CATCCGTAAAGACCTCTATGCCAAC-3' |
| Actin-R | RT-PCR | 5'-ATGGAGCCACCGATCCACA-3' |

[a)]F, forward; R, reverse.

**Table S2.** Antibodies used in this study.

| Antigen | Clone number | Supplier |
|---|---|---|
| β-actin | AC-15 | Sigma |
| CD31 | MEC13.3 | BD |
| APC-CD11b | M1/70 | eBioscience |
| Alexa Fluor 488-F4/80 | BM8 | eBioscience |
| Purified F4/80 | BM8 | eBioscience |
| Biotin-Ly6G | 1A8 | eBioscience |
| CD206 | 15-2 | eBioscience |
| APC-Ly6C | HK1.4 | eBioscience |
| PE-avidin | | eBioscience |
| BV421-CD45 | 30-F11 | BD |
| PE-CD4 | RM4-5 | BD |
| APC-CD8 | 53-6.7 | BD |
| FITC-CD3 | 17A2 | BD |
| Ki67 | SP6 | Lab Vision |
| Annexin V | | eBioscience |
